# Ultra-high throughput single-cell analysis of proteins and RNAs by split-pool synthesis

**DOI:** 10.1038/s42003-020-0896-2

**Published:** 2020-05-07

**Authors:** Maeve O’Huallachain, Felice-Alessio Bava, Mary Shen, Carolina Dallett, Sri Paladugu, Nikolay Samusik, Simon Yu, Razika Hussein, Grantland R. Hillman, Samual Higgins, Melanie Lou, Angelica Trejo, Laura Qin, Yu Chuan Tai, Shigemi M. Kinoshita, Astraea Jager, Deval Lashkari, Yury Goltsev, Sedide Ozturk, Garry P. Nolan

**Affiliations:** 1 Roche Sequencing Solutions, Pleasanton, CA USA; 2 Apprise Biosciences, Menlo Park, CA USA; 3 Department of Microbiology and Immunology, Baxter Laboratory for Stem Cell Research, Stanford University School of Medicine, Stanford, CA USA

**Keywords:** Immunogenetics, Diagnostic markers, Proteomic analysis, Tumour immunology, Transcriptomics

## Abstract

Single-cell omics provide insight into cellular heterogeneity and function. Recent technological advances have accelerated single-cell analyses, but workflows remain expensive and complex. We present a method enabling simultaneous, ultra-high throughput single-cell barcoding of millions of cells for targeted analysis of proteins and RNAs. Quantum barcoding (QBC) avoids isolation of single cells by building cell-specific oligo barcodes dynamically within each cell. With minimal instrumentation (four 96-well plates and a multichannel pipette), cell-specific codes are added to each tagged molecule within cells through sequential rounds of classical split-pool synthesis. Here we show the utility of this technology in mouse and human model systems for as many as 50 antibodies to targeted proteins and, separately, >70 targeted RNA regions. We demonstrate that this method can be applied to multi-modal protein and RNA analyses. It can be scaled by expansion of the split-pool process and effectively renders sequencing instruments as versatile multi-parameter flow cytometers.

## Abstract (web summary)

Maeve O’Huallachain et al. report a method that enables simultaneous, ultra-high throughput single-cell barcoding for targeted single-cell protein and RNA analysis. They show the utility of their method in analyses of mRNA and protein expression in human and mouse cells.

## Introduction

Until recently single-cell analysis has been primarily the provenance of flow cytometry, enabling strides in constructing our understanding of the immune system, cancers, and other complex cell systems^1–4^. This has resulted in an extraordinary history of clinically actionable benefits to patients—from understandings of HIV-1 infection to auto-immune processes and cancer immunotherapy to name a few. The goals for advances in flow cytometry are clear: measure as many relevant target molecules per cell as quickly as possible. This goal is accomplished by tagging an antibody with a uniquely identifiable agent that is a surrogate for the level of expression of a given cellular constituent.

Fluorescence and isotope tagging are the principal means for measuring antibody binding to cells in flow cytometry. Fluorescence-based flow cytometry enables the highest cell throughput (20–30,000 cells per second) of any technology thus far but is limited by the number of simultaneous parameters that can be determined per cell (10–15 by accomplished groups) with up to 28 possible reported in public conferences^5,6^. Mass cytometry (CyTOF), currently enables up to 50 parameters to be measured simultaneously at a rate of 500–1000 cells per second^7–10^. Mass cytometry overcomes limitations of spectral overlap and auto-fluorescence inherent to fluorescence-based measurements. Both technologies have been primarily focused on measurement of protein epitopes, but have been used to measure nucleic acids such as targeted mRNA^11,12^. Both fluorescence and isotope tagging are currently limited by the number of unique tags.

Single-cell sequencing has made considerable progress relying on the notion that the sequence is the tag. Single-cell RNA-seq^13–19^, whole genome, and open chromatin analyses have brought us lineage mapping of cancer, differentiation, immune cell profiling^20–22^, and RNA expression stochasticity^23–26^. Published reports of between one to a few thousand cells in a given experiment are reported. Multiplexing by single-cell barcoding of transcripts is accomplished by manipulations of single cells into microwells (sorting)^13,19,24,25^, nanowells^27^, or oil-encased microdroplets^28,29^. Sorting is limited by microwell aiming accuracies. Microdroplets can barcode more cells but worries persist about the differential chemistry and resulting biases accomplished in each droplet. Both of these latter techniques risk changes to the metabolism during cell preparation and barcoding prior to cell lysis– since both approaches, generally, rely on the use of live cells^30^. Commercial approaches have costs ranging from $1 to $5 per cell per expressome.

The objective has been the same across all these technologies: more information on more cells at the lowest cost, balanced against the requirements for precision measurement and an attendant need for tailored bioinformatics^31^. Approaches that enable simultaneous measurement of protein expression levels (via antibody binding) and RNA expression is desired—since in many situations this can lead to a more holistic view of given cellular processes^32^. An efficient merging of these techniques onto a single, expansible, platform would allow for more cells to be measured with more parameters representing diverse biology. Microdroplet-based approaches have added protein analysis to the existing transcriptome analysis enabled by the droplet techniques^32,33^, however they remain limited to a few thousand cells per consumable chip with costs out of reach for many laboratories. Sequence-based tagging^34,35^ with error correction^36^, of antibodies can greatly expand the number of parameters measurable; theoretically billions of distinct tags could be created.

We here present an approach for linking cell-specific barcodes to objects on or within a given cell using a variant of traditional split-pool synthesis. Our process, presented previously^37^, builds a cell-specific barcode from multiple individual subcodes dynamically on all targets in a given cell in a stepwise process. The result is that every labeled cDNA in a cell, or antibody bound to a cognate target, is labeled with a cell-specific combination of subcodes. We demonstrate simultaneous tagging of several million cells in this manner and sequencing of ~50,000 of such cells per experimental run (a limitation only imposed by sequencing yield). We demonstrate accurate measurement of up to 50 antibodies simultaneously for protein analysis, up to 73 regions of 16 mRNA targets simultaneously, and 1 antibody combined with 40 regions of 13 mRNA targets for simultaneous multimodal protein and RNA analysis. Similar combinatorial indexing approaches were previously applied to single cells and nuclei for analyses of transcriptomes, genomes, methylation, and chromosome accessibility and conformation^38–44^. Our study is the first demonstration to our knowledge of protein analysis by combinatorial indexing of single cells via oligonucleotide barcoding. This approach enables barcoding and analysis of millions of cells in high-parameter space at low cost compared with other single-cell protein analysis technologies. We demonstrate single-cell combinatorial indexing of commercially available oligonucleotide-conjugated antibodies sold for use in CITE-seq, which can provide substantial cost-savings over droplet-based methods. As single-cell transcriptome coverage is typically sparse per cell, we opted to target specific RNAs to enable higher coverage for regions of interest and combine targeting of RNA and protein modalities on the same cells. The approach complements other whole transcriptome sequencing systems, could enable a new form of gating by RNA sequence, and provides an approach to render sequencing instruments as effective substitutes for traditional flow cytometry platforms. Building this technology towards commercial use with concomitant standardization will allow this technology to provide a higher throughput alternative to current single-cell analysis techniques.

## Results

### Overview

**Fig. 1:**
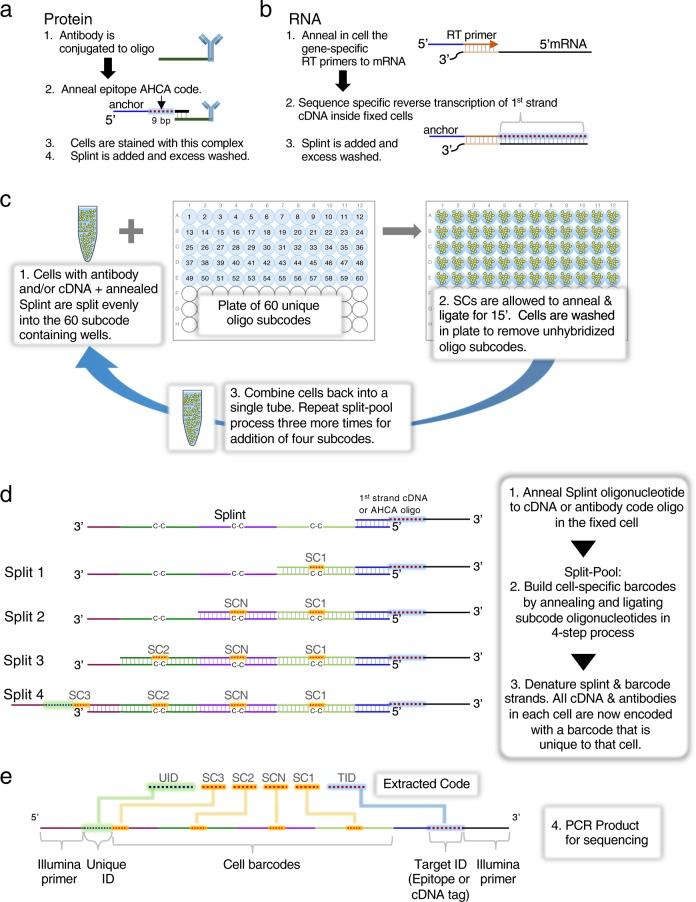
Methodology and Schematics of Single-cell Barcoding by Split Pool. **a** Method for single-cell QBC antibody coding. Antibodies are conjugated to a linker oligonucleotide. An AHCA barcode oligo is annealed to the linker oligo prior to staining the cells with the antibody-conjugate annealed to AHCA oligo complex. This AHCA oligo also contains an “anchor” sequence for later annealing of the Splint. Cells are stained with pools of antibodies prepared this way. **b** Method for single-cell QBC mRNA coding by reverse transcription. A panel of gene specific primers is added to fixed cells and reverse transcription is initiated. Splint is added via annealing to the complementary anchor region on the reverse transcription initiator. **c** Split-pool steps for quantum barcoding. Step 1. Cells are randomly split into 60 wells of a 96-well microtiter plate each of which contains one of 60 unique short oligonucleotides. Step 2. The SC subcode anneals to the Splint and is ligated to the adjacent sequence. Cells are washed of free SC. Step 3. Cells are pooled and allowed to go through the split-pool process with successive sets of SC oligos. The process is repeated a total of four times. **d** Schematic description of general QBC method. The respective reverse transcribed or antibody bound probes from 1a-b in cells are treated with the Splint sequence. The Splint sequence binds to the cognate oligo in the cell. Excess Splint is washed away. The cells are subjected to the split-pool process outlined in Fig. 1c. The codes are annealed on the Splint and ligated in place to link the structure together. To avoid mismatches at the barcode region, the Splint oligo has carbon spacers (C-C) to allow different barcode sequences to anneal. At the end of the split-pool process, the cells are lysed and DNA prepared for PCR. **e** Final structure of libraries to be sequenced. Post PCR, the final structure of the material(s) are shown representing either the presence of antibodies or a given targeted mRNA. At the top of the panel are the extracted code sequences removed with the parsing program.

We employ the classic split-pool^37,45,46^ process for assembling cell-specific barcodes on molecular targets, protein or RNA (Fig. 1a–e). For proteins, a universal common oligonucleotide sequence is conjugated to antibodies. This common oligonucleotide anneals to a second oligonucleotide with a 9 bp unique barcode identifier, termed Assayable Hybridization Code Adapter (AHCA). The AHCA also contains a region, termed the anchor, complementary to the Splint oligonucleotide. The Splint is used to position the growing subcodes for ligation during the split-pool process. Antibodies are prepared individually (Fig. 1a, steps 1 and 2) and mixed into user defined panels prior to staining of cells. For RNA, (Fig. 1b) fixed and permeabilized cells are in situ reverse transcribed with gene specific primers that contain the same anchor sequence.

The split-pool process is initiated by evenly dividing up any given starting number of cells (we routinely barcode 1–20 million cells, though the process can be scaled in a variety of manners) into each of 60 wells of a plate (Fig. 1c). In each well, there is present one of 60 distinct subcodes (SC1-1 through SC1-60). The subcodes contain flanking regions designed to specifically anneal the SC to a region on the Splint (Fig. 1d). Between the regions that anneal to the Splint, is an intervening unique 7-mer (containing a Levenshtein error-correcting code^36^). The 7-mer code has no corresponding complementary region in the Splint, but rather a 3 carbon backbone linker designated C–C. The C3 linker minimizes preferential binding of any one subcode and prevents SC sequences from acting as primers on the Splint during PCR. After annealing the SC1 oligonucleotides to the Splint, adding ligase will covalently connect the 3′ termini of each SC1 to the antibody tag or cDNA. The cells from all of the 60 wells are then pooled to randomize them. See Supplementary Data 1–11 for oligo and primer sequences.

By repeating the process three more times, each of the 1 million cells will have one of 60^4^ = 12,960,000 possible subcode combinations. Each extender-enabled object (antibody and/or cDNA) in each cell has the same cell-specific combination of subcodes because all objects in a cell travelled through the same random synthesis path during each of the splits. Each split-pool experiment can be readily managed on four 96-well plates (one per split-pool round). The schematic of a generic, completed split-pool product (comprised of each of the 7 bp subcodes with a coordinate target ID) is shown in Fig. 1e. Each sequence that represents either a protein or RNA target is termed a quanta. Hence, we named the entire process Quantum Barcoding (QBC).

The probability of any two cells following the same path through the splits can be calculated. For instance, allowing 5% error that any two cells will have the same complete barcode, as many as 500,000 cells can be barcoded using four splits and 60 oligonucleotides per split. The solution is computed using a generic application of the birthday paradox and is detailed in Supplementary Table1 ^47^. Experiments in this report are targeted to a goal of 12,900,000 barcodes which would give an expected duplicate cell barcode rate of 0.2% or 96 repeated cell codes per 50,000 cells. The number of splits and subcodes for any given probability and cell population size can be modified depending on the scale of the experiment. At the end of the split-pool process, Illumina sequencing adapters are added by PCR amplification, libraries are sequenced, and the data is informatically deconvoluted into single-cell codes as shown in Fig. 1e. This data is used to assemble cell-based representations of target molecules on a single-cell basis. We have devised a filtering strategy of reads associated with single-cell barcodes that requires a minimum number of markers and reads to identify intact single cells.

### Multiparameter single-cell barcoding of proteins

We conjugated seven antibodies (TCRb, CD3, CD4, CD8, IgM, CD44, and B220) to oligonucleotides and annealed a specific AHCA-extender sequence to each per the above procedure. This antibody mix was used to stain fixed murine spleen cells. The split-pool procedure was carried out followed by library preparation PCR and MiSeq sequencing on an aliquot representing ~50,000 cells (estimated by image-based counting of an aliquot of cells). As a control, we stained a parallel splenic cell mixture with the same antibodies that had been conjugated to traditional fluorophores.

To rapidly decode the millions of sequences generated in a single experiment, assign them to cells, and count the epitope (AHCA) sequences, we developed a software pipeline that rapidly processes up to hundreds of millions of sequence reads, deconvolutes, and deduplicates them into single-cell counts of the markers (protein and/or RNA) within each cell. Metrics for sequencing, cell counts, and analysis filters for each experiment are presented in Supplementary Data 12. The number of cells detected in the data averaged over 24 experiments presented in this report was ~41,000, closely matching the estimated 50,000 cell number input into each library preparation PCR (implying we are measuring every cell event). The data were processed into FCS file format, uploaded to a standard flow cytometry analysis platform (Cytobank, Inc.)^48^, and the data displayed in conventional 2D contour plots. Expression is represented on ArcSinh plots^49^ wherein each axis represents the number of unique sequences counted per epitope tag for each cell. See Supplementary Data 13 for file names and plot settings. For the majority of data representations in this report, the data is normalized to total sequence counts per cell.

**Fig. 2:**
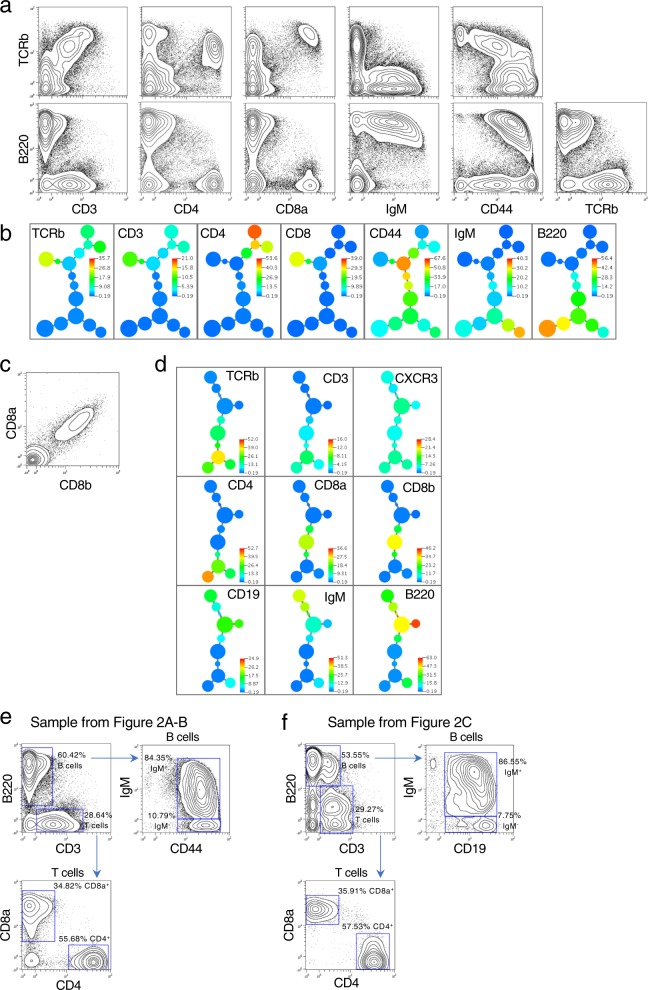
Single-cell expression profiling of protein epitopes by quantum barcoding. **a** Wildtype murine spleen cells were stained with seven antibodies. Sequence data was converted to an FCS file for display in 2D contour plots. In all, 63,870 cells were detected by the analysis and are represented in the plots. See Supplementary Figure 1 for biaxial plots for all markers. **b** An X-shift derived minimum spanning tree is shown for the data used in Fig. 2a. The tree is colored in a heatmap format per the marker shown in each panel. Appropriate co-expression of markers is seen for T cells (including a split for CD4 and CD8) as well as B cells. All seven markers were used in the clustering run. **c** Co-detection of epitopes on the CD8 heterodimer. Multiple antibodies (CD8a, CD8b, TCRb, CD4, CD3, B220, CD19, IgM, CXCR3) were used to stain murine splenic cells. Antibodies were used that are specific to two distinct epitopes present on the heterodimeric chains of CD8 (designated CD8a and CD8b). In all, 21,307 cells represented. The data displayed in a biaxial plot is unnormalized to highlight the correlation between the two CD8 subunits. Normalized biaxial plots of all markers are in Supplementary Fig. 2. **d** An X-shift driven minimum spanning tree is shown for the data in Fig. 2c for all the markers used in this staining. All nine markers were used in the clustering run. Data is normalized. **e** Phenotyping murine spleen cell populations by traditional bivariate gating. Two murine splenic cell samples shown in Fig. 2a–d were gated for T and B cell subsets. B cells were identified by the presence of B220 and T cells by the presence of CD3. Additional markers (IgM, CD44, CD19, CD8a, and CD4) were used to further subset the T and B cell populations. The two samples gated in this manner had comparable cell percentages by this gating strategy.

Figure 2a shows split-pool tagged antibodies against seven unique proteins. The data shows all the features of a standard FACS analysis for the markers used. Additionally, to visually relate co-marker expression, we used the previously developed (by NS and GPN) X-Shift algorithm^50^ (Fig. 2b). In concept, it is a deterministic variant of our previously published SPADE algorithm^51^ in which cells are clustered by a density determination algorithm and the clusters linked in a minimum spanning tree (MST). Co-expression of T cell specific markers (TCRb and CD3) are coincident with CD4 or CD8, whereas CD4 and CD8 are not co-expressed, as expected. B220 (the antibody of which binds at extremely high levels) and CD4 markers are non-coincident, and B220 co-expresses with IgM each into the second decade of expression levels, as expected. CD44, a T cell activation marker of antigen experience, showed variable expression and demarcated cell subsets for activated CD8 and CD4^52,53^.

We demonstrated the correlative capabilities of co-staining using two markers known to co-exist on the same protein. The CD8 surface protein expressed on T cells is composed of a heterodimer, CD8a and CD8b. Antibodies distinctly able to bind epitopes on each monomer were conjugated with the QBC common oligonucleotide and given unique AHCA sequences. When CD8a and CD8b were plotted against each other in a non-normalized plot (Fig. 2c) a near perfect correlation of expression is observed, demonstrating succinctly the capability of the QBC process to label co-expressed proteins in single cells. The X-shift derived MST is shown in Fig. 2d for all 9 antibodies co-stained with the CD8a and CD8b antibodies in this experiment.

We performed traditional bivariate gating (Fig. 2e, f) of the two experiments shown in Fig. 2a–d. Using biaxial plots, we gated the T and B lymphocyte populations observing similar lymphocyte subtype percentages between the two samples. These percentages fall in the expected ranges for a murine splenic cell type.

**Fig. 3:**
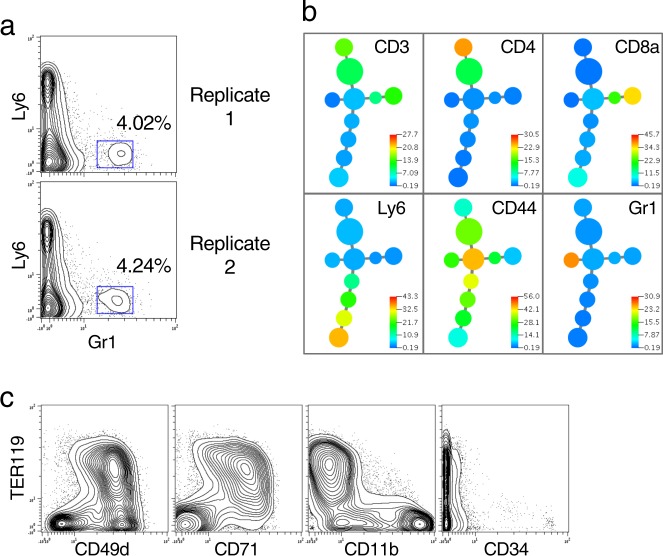
Detection of a rare cell population. **a** A comparable experiment to those in Fig. 2 was carried out on mouse spleen cells using 6 antibodies to CD3, CD4, CD8, CD44, Ly6, and Gr1. Duplicate stains and split pools were run by two individuals (GPN and MS). The cell population representing the rare Gr1+ cells are on the right side of each panel. Biological replicates 1 and 2 represent 9281 and 9715 cells, respectively. Biaxial plots of all markers are in Supplementary Fig. 3a, b). **b** An X-shift driven minimum spanning tree is shown for the data in Fig. 3a replicate 1 for all the markers used in this staining. All six markers were used in the clustering run. MST for both replicates shown in Supplementary Fig. 3c, d. **c** Staining of Bone Marrow for erythroid markers. 12,373 bone marrow cells were detected with oligo-conjugated antibodies specific for TCRb, B220, CD11b, CD34, CD49d, CD71, TER119, CD135, and CD41. Biaxial plots for all markers are in Supplementary Fig. 4.

We determined if rare subsets of cells could be discerned with coefficients of variation (CVs) comparable to that seen by traditional cytometry. In Fig. 3a, two markers (Ly6 versus Gr1) are shown that demarcate dendritic cell subpopulations out of a set of 6 protein targets analyzed on a murine spleen cell sample. Distinct subpopulations of Gr1 positive cells at expected frequencies (observed in ~4% of cells) can be clearly observed in the FACS-like single-cell format derived from sequencing data. The X-shift derived MST is shown in Fig. 3b, further demonstrating the unique cell populations that can be readily identified via QBC single-cell analysis of protein expression.

This latter result implies the cell population observed can be measured at a level of 380 cells (4% of ~9500). Individual cells can be clearly observed, meaning the definition rate of cells which have sufficiently unique markers can be observed at the rate of 1 in 9500 or 0.01%. Note that the accuracy of the measurements depends upon mutual expression of multiple unique markers because increasing the dimensionality means that background binding events become lost in the high dimensional space. The only cells that are found by the clustering algorithm are those that create sufficient density in N-space to be mathematically detected above a given threshold. This principle has been used in stem cell research with multiple required co-expressed markers and so-called lineage minus dump channels that remove cells that express mature lineage markers^54^.

**Fig. 4:**
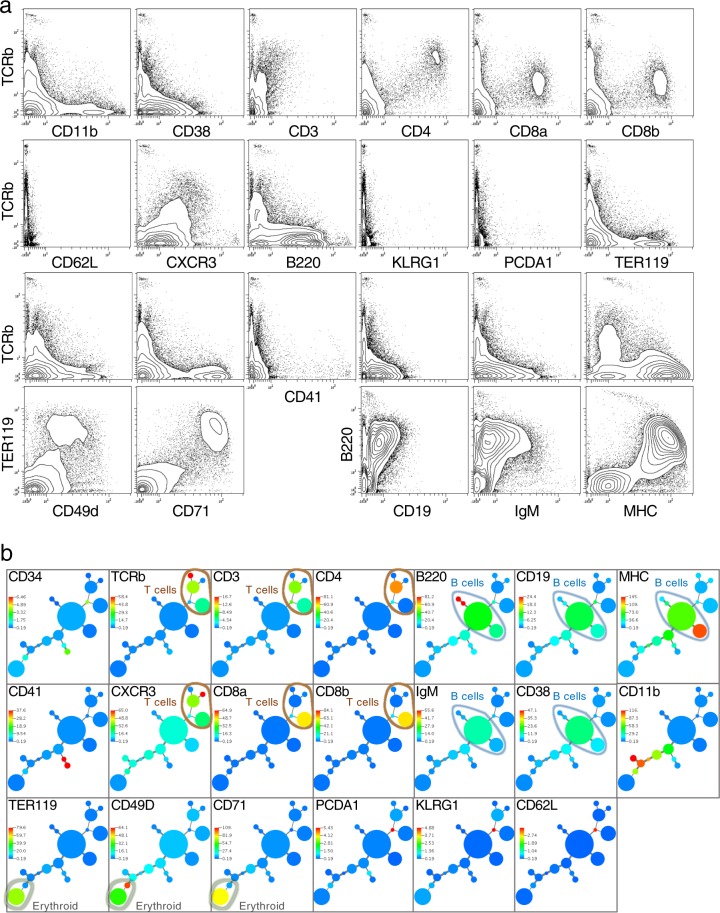
Multiparameter single-cell protein barcoding. **a** In all, 20-parameter antibody staining demonstrates appropriate co-expression of markers. A 50:50 mixture of splenic and bone marrow cells was analyzed with QBC and the analysis outcome representing 34,150 cells plotted. Expression profiles match cell expression patterns and proportions expected for these murine organs. Antibodies used in this staining: TCRb, CD3, CD4, CD8a, CD8b, MHC, CD62L, B220, IgM, CD19, CD38, CD11b, CXCR3, CD34, PCDA1, CD49d, CD71, TER119, KLRG1, and CD41. Biaxial plots for all markers are in Supplementary Fig. 5. **b** Automated clustering and MST representation of a mixed population of bone marrow and splenic cells demonstrates appropriate cell subsets and groupings. An X-shift driven minimum spanning tree is shown for the data in Fig. 4a for all the markers used in the staining. T cell markers that co-express on the same cell subsets (TCRb, CD3, CD4, CD8, and CXCR3) are circled with a light brown line. B cell markers that co-express (B220, IgM, CD19) as well as MHC and CD38 known to express on B cells are circled with a light blue line. Erythroid marker co-expression of TER119, CD49D, and CD71 is circled with a light gray line.

We applied an antibody panel against nine protein targets to another murine cell type, bone marrow (Fig. 3c). These markers correctly called out erythrocyte marker co-expression (TER119, CD49d, and CD71). Critically, even rare CD34 positive cells could be discerned at a frequency commensurate with their normal expression. This 9 antibody panel was added to a 14 antibody panel suitable for spleen to stain a 50:50 mixture of splenic and bone marrow cells creating a cell mixture appropriate for a 20-parameter antibody stain. Figure 4a displays the data which demonstrates expected patterns of expression for the cell populations, co-expression of expected proteins, and expected anti-correlations of protein expression. An MST tree was created for the combined panel of 20 antibodies (Fig. 4b). Cell clusters discerned by X-shift showed expected co-expression patterns. Several relevant branches of the tree (T cells, B cells, erythroid cells) are shown. While the MST tree does call out differing cell subsets, it should not be mistaken for a map of differentiation. Different algorithms that can more subtly detect progression changes in marker expression^10,55,56^ are required for such representations.

**Fig. 5:**
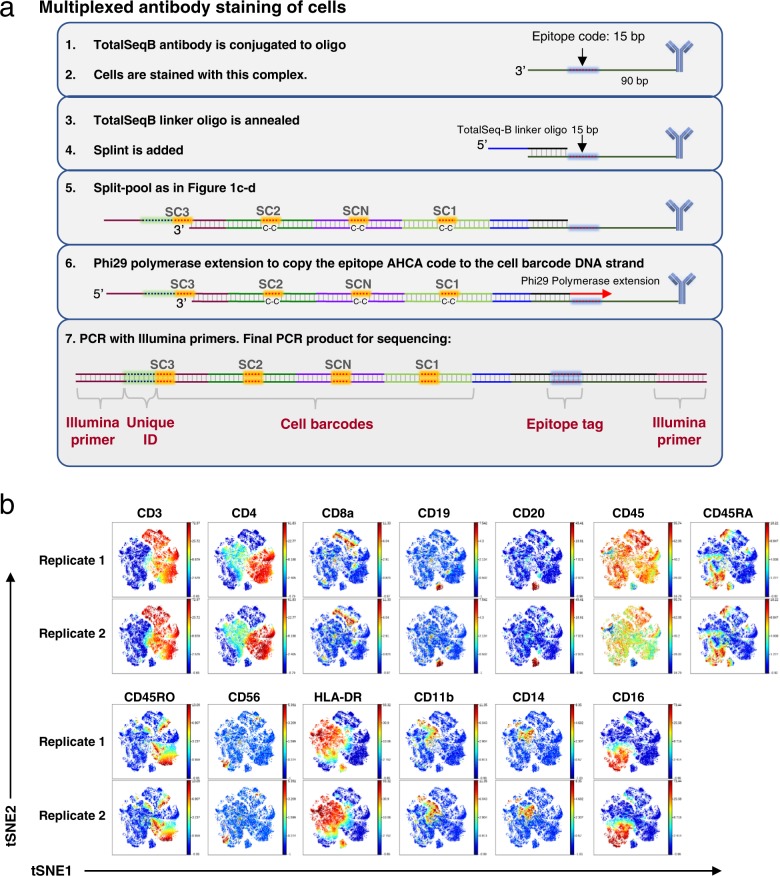
Protein analysis in human cells. **a** Schematic of anti-human antibody QBC workflow. (1–2) TotalSeq-B antibody-oligonucleotide conjugates are stained on cells. (3) A TotalSeq-B linker oligonucteotide is annealed to the oligonucleotide conjugated to the antibodies which contains the “anchor” sequence for later annealing of the Splint. (4–5) Split-pool is done as in Fig. 1. (6) A Phi29 polymerase extension copies the epitope code to the cell barcode DNA strand. (7) PCR with Illumina primers is done to prepare the library for sequencing. **b** viSNE plots for data from the biological replicates of a 13 anti-human antibody panel applied to human PBMCs. viSNE clustering using 12 out of the 13 markers. CD45 was left out of the clustering analysis to observe how the other leukocyte markers correlated with each other.

The emergence of commercially available anti-human antibody-oligonucleotide conjugates marketed for use with CITE-seq^32^ by BioLegend provided the opportunity to demonstrate even higher parameter protein analysis on human cells. The TotalSeq-B antibodies could be quickly adapted to the QBC workflow with a few minor changes because they contained the same Illumina primer-binding sequence used for our murine cell experiments. Figure 5a shows the workflow adapted for TotalSeq-B antibodies. A TotalSeq-B linker oligonucleotide was designed with complementarity to the 3′ end of the TotalSeq-B-conjugated oligonucleotides and complementarity to the 5′ end of the QBC Splint oligonucleotide. Cells were stained with pools of TotalSeq-B antibodies and incubated with the TotalSeq-B linker oligonucleotide before proceeding with split-pool as in Fig. 1. After split-pool a polymerase extension was needed to copy the epitope tag sequence to the same strand as the QBC subcodes before PCR.

We initially tested panels of 13 (Fig. 5b and Supplementary Fig. 6) and 32 (Supplementary Figs. 7 and 8) TotalSeq-B antibodies on healthy human PBMCs with two biological replicates for each panel size. Analysis of the sequencing data yielded 47,081 and 92,016 cells for the 13 antibody panel replicates and 92,788 and 82,478 cells for the 32 antibody panel replicates. The expected versus observed cell count discrepancy likely derives from error in cell counting, aliquoting by pipetting, and background in the system due to non-specific binding of antibodies or oligos. Using traditional bivariate gating and viSNE^57^ visualization we observed the expected co-expression patterns of the PBMC cell populations^58,59^.

**Fig. 6:**
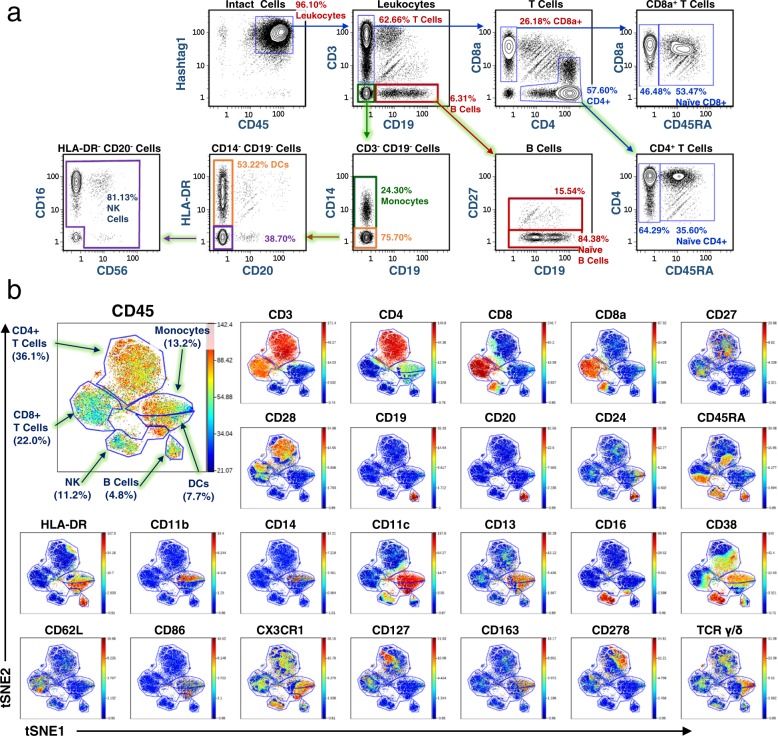
Human PBMC population phenotyping. **a** Phenotyping of healthy human PBMC populations using surface markers targeted using a 50-parameter antibody panel. Human PBMCs were stained with 50 TotalSeq-B antibodies and barcoded by QBC. 42,165 cells identified by data analysis of the ~26 M sequence reads. Immune cell populations were gated by traditional bivariate gating. Expression profiles and percentages are within the expected ranges for this cell type. **b** Expression of PBMC cell populations demonstrated via viSNE plots demonstrates appropriate cell subsets and groupings. A viSNE analysis was done for the data in Fig. 6a and a subset of the plots are shown. See Supplementary Fig. 9 for the complete set of viSNE plots. In all, 50 TotalSeq-B antibodies were used in the experiment: CD3, CD4, CD8, CD8a, TIGIT, CD274, CD27, CD28, CD25, CD137, CD19, CD20, CD24, CD45, CD45RA, CD45RO, CD56, CD69, HLA-DR, CD11b, CD14, CD11c, CD13, CD197,CD206, XCR1, CD15, CD16, CD38, CD62L, CD86, CD80, CX3CR1, CD127, CD158b, CD163, CD278, CD279, CD314, CD335, CD10, CD57, TCR α/β, TCR γ/δ, TCR Vγ9, CD155, Hashtag 1, IgG1, IgG2a, and IgG2b. viSNE analysis was done using 46 out of the 50 markers. Hashtag 1 antibody and the three isotype IgG control antibodies were left out of the clustering analysis to observe how the anti-human leukocyte markers correlated with each other.

Next, we applied 47 anti-human antibodies and 3 isotype controls available in the Biolegend TotalSeq-B configuration to healthy human PBMCs for QBC analysis. Traditional bivariate gating of T and B lymphocytes, monocytes, DCs, and NK cells were comparable to expected proportions in PBMCs (Fig. 6a)^58,59^. The viSNE dimensional reduction algorithm was applied and cell subtypes identified and gated by marker co-expression (Fig. 6b).

Thus, limited only by sequencing depth, QBC can effectively demarcate expression of protein markers at a parameterization similar to CyTOF using a high-throughput DNA sequencing instrument. This, in effect, renders sequencing instruments as high throughput single-cell cytometers.

### Single-cell barcoding of targeted mRNA

It would be advantageous to extend the QBC approach developed here to RNA expression measurements at the single-cell level. Two approaches were investigated for their relative efficiency towards various goals relevant to RNA expression in cells. The first approach, termed “In situ RT-QBC” (InsRT-QBC), initiates in-cell reverse transcription using oligonucleotides to targeted sequences within genes of interest. We previously applied a more traditional variation of proximity ligation and RCA^12^ (AB, NS, GPN) to enable multi-parameter mRNA expression analysis at the single-cell level by fluorescence-based and CyTOF-based flow cytometry. The second approach, termed “Specific Nucleic Acid detection via Intramolecular Ligation (SNAIL) and Rolling Circle Amplification (RCA)” (SNAIL-RCA) uses a structural adaptation of the padlock probe/proximity ligation/rolling circle amplification process^60,61^. The InsRT-QBC enables direct sequencing of mutations or variations of intron/exon usage, while the SNAIL-RCA technique can amplify the signal from RNA present at lower levels of relative expression.

**Fig. 7:**
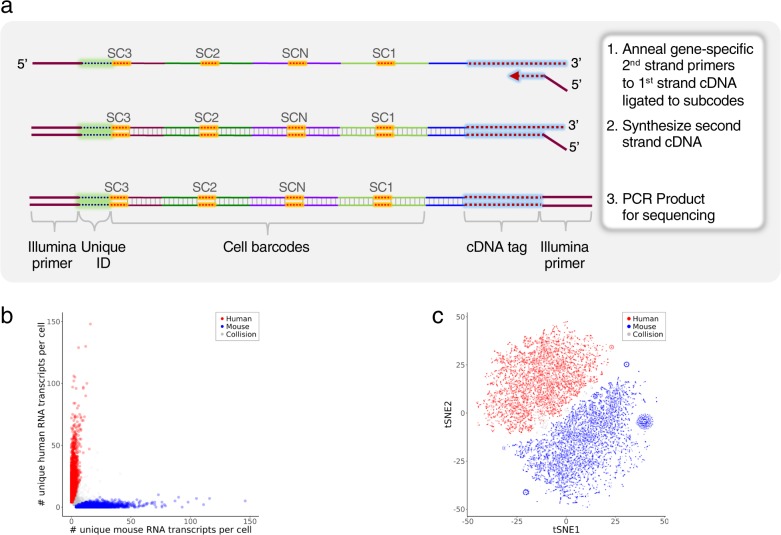
Targeted mRNA QBC workflow. **a** Schematic of targeted mRNA QBC workflow. Post lysis of cells analyzed for mRNA, a second sequence specific primer that contains the second Illumina primer sequence is used to synthesize the completed strand. The remaining mixture is PCR amplified for Illumina sequencing. **b** Single-cell expression of human and mouse transcripts in a 50:50 mixture of human Nalm6 and mouse BW5147 cells. RNAs targeted: ACTB, CD19, CD3E, CD4, CXCR4, HINT1, HSP90AB1, LAT, LCK, NAP1L1, OAZ1, PLCG1, PTPRC, ZAP70. The three cell aliquots sequenced had ~102, ~90, and ~38 M raw reads from which 25,610, 15,148, 12,499 cells were identified, respectively. Scatter plot of the number of unique RNA transcripts of each species type per cell for the 25,610 cell sample. Cells were color coded as human (red, 44%) or mouse (blue, 52%) if ≥75% of the total unique transcripts were from one of the species, otherwise the cells were labeled as collision (grey, 3.6%). Two outliers that had >150 unique transcripts per cell were not included in the plot. Two additional cell aliquots are shown in Supplementary Fig. 10a. **c** tSNE clustering of the mixture from Fig. 7b. Normalized data were visualized by t-SNE. Cells were colored as human (red), mouse (blue) and collision (gray) based on the cluster-assignment inferred from the number of unique RNA transcripts per cell as described in Fig. 7b. Two additional cell aliquots are shown Supplementary Fig. 10b.

For InsRT-QBC, 1–5 primer pairs were designed per transcript. Comparable to the work of Church and colleagues^62^, to increase cellular retention of the reverse transcription product, amino-allyl dUTP is included during in situ reverse transcription followed by crosslinking with an amine-reactive crosslinker, DTSSP. After reverse transcription, the cells were treated per Fig. 1c, d to add Splint and process the cells through split-pool barcoding. After cell lysis, the cognate targeted second strand primer is used along with Illumina primers to amplify the final product (Fig. 7a).

InsRT-QBC primers were designed to measure the relative expression of multiple mRNAs in a mouse cell line and several human cell lines. A 50:50 mixture of mouse T cell line, BW5147, and human pre-B cell line, Nalm6, were processed by InsRT-QBC to demonstrate the specificity of the assay. In all, 58 targeted primers to 14 RNA targets were used for reverse transcription. Following split-pool cell barcoding of the cell mixture, three aliquots of 50,000 cells were processed for library preparation and sequenced on NextSeq. The numbers of mouse unique transcripts versus human unique transcripts per cell for one aliquot were plotted (Fig. 7b) showing effective separation of the cell line types. The data was visualized with the tSNE algorithm^63^ (Fig. 7c). The plots show clear separation of the two cell types after dimensional reduction. Collision cell barcodes can result from physical cell doublets, barcode duplications, or RNA diffusion between cells. We observed a low rate of these multiplets based on the mouse and human mixture. The three aliquots gave comparable cell type separation.

**Fig. 8:**
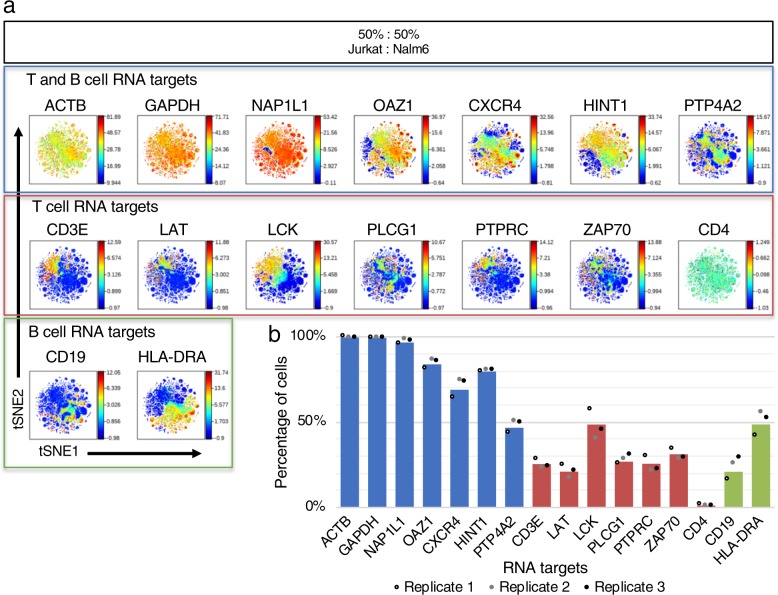
Single-cell expression clustering of T and B cell signaling genes. **a** RNA expression of T cell and B cell signaling protein-coding genes demonstrated via viSNE plots of a 50:50 mixture of Jurkat and Nalm6 cell lines. Data from an InsRT-QBC experiment was rendered via viSNE using 14 of the 16 targets as selected channels to organize the viSNE plots. The housekeeping markers GAPDH, ACTB were deliberately excluded from the viSNE run to view how the expression of the other markers correlated with each other. Anti-expression of the T and B cell markers is clearly visible. Plots represent 51,760 cells. Two additional biological replicates are in Supplementary Fig. 11. **b** Bar graph of the percentage of cells expressing each RNA target from *n* = 3 biologically independent cell samples. Single-cell bivariate plots were hand gated for cells positive for the RNA targets. The average percentages for the three replicates were plotted as bars and individual data points for the three replicates were overlaid. RNA targets only expected to be expressed in T cells (red) or B cells (green) have percentages at or below ~50% while all but one RNA target expected to be expressed in both cell types (blue) are detected in >50% of the measured single cells. The housekeeping markers, GAPDH and ACTB, were measured in ~99% of cells.

We tested human cell line mixtures of 50:50 Jurkat T cell line and Nalm6 pre-B cell line by InsRT-QBC targeting 16 RNA transcripts with 73 primer pairs. Three 50:50 cell mixture biological replicates were carried out on three different days by two different operators (MO and LQ). Single-cell expression counts from the three experimental replicates were clustered together by viSNE analysis (Fig. 8a). Cell populations were hand gated and the percentage of cells positive for each targeted RNA was plotted (Fig. 8b). The percentages were consistent with the expectations for a 50:50 mixture of the cell types. Similar to other single-cell analysis platforms, there are dropouts for individual RNA targets in many cells. The dropouts here may be due to RNA degradation or leakage prior to conversion to cDNA, inefficiencies of the in situ reverse transcription and cell barcoding, or sequencing depth. While cell lines are thought to be homogeneous, expression variability across single cells may also contribute to the absence of signal for some RNA targets.

**Fig. 9:**
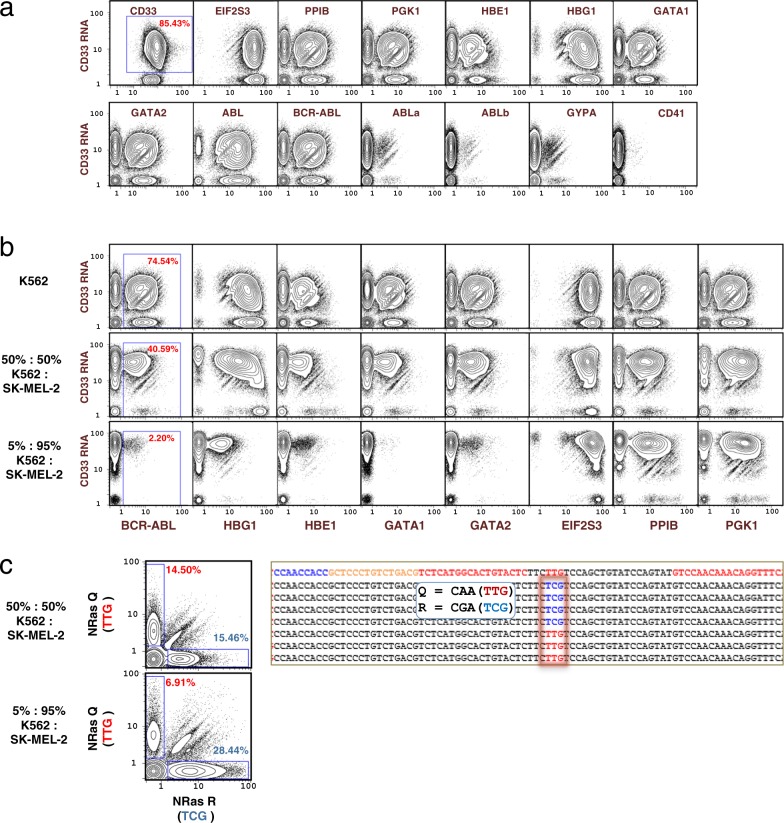
Application of single-cell barcoding of targeted mRNA. **a** Single-cell expression of 14 RNA transcripts by InsRT-QBC and 1 protein surface marker in K562 cells. BCR-ABL primers flank the fusion junction while ABLa and ABLb primers flank the non-fusion ABL isoforms at the fusion junction. ABL reverse transcription primers target transcript sequence downstream of the fusion junction. The library was sequenced on a HiSeq that yielded ~179 M reads for the sample from which 56,950 single cells were identified. CD33 protein expression level was much higher than any of the RNA target levels and was not included in the normalization of the RNA targets. The complete set of biaxial plots can be found in Supplementary Fig. 12. **b** Detection of RNA expression in cell mixtures. Comparison of 3 cell mixtures for single-cell expression of nine mRNA transcripts demonstrates differential expression between K562 cells and SK-MEL-2 for transcripts specific to blood cells (BCR-ABL, HBG1, HBE1, GATA1, GATA2) while expression from EIF2S3, PPIB, PGK1, and CD33 remains consistent between the mixtures. The BCR-ABL oncogene, is shown with the expected diminution when K562 cells were diluted with SK-MEL-2 cells. Plots represent 56,950 (K562), 54,085 (50:50 mixture), and 60,754 (5:95 mixture) cells. The complete set of biaxial plots can be found in Supplementary Figs. 12–14. **c** Distinct detection of NRAS SNP alleles by QBC. Mixtures of SK-MEL-2 cells and K562 cells were subjected to QBC. Plots of expression detected from the NRAS transcript at a known SNP location in the SK-MEL-2 cell line show largely mono-allelic expression at the locus, with a slight preference for expression of the R allele. Representative sequences are shown on the right where the wildtype Q allele at position 61 is shown as red (TTG) and the mutant R allele is shown as blue (TCG). The top line of the sequence represents a generic sequence for alignment. The flanking red sequences were the gene specific regions used in the primers. Plots represent 54,085 (50:50 mixture), and 60,754 (5:95 mixture) cells. The complete set of biaxial plots can be found in Supplementary Figs. 13 and 14.

InsRT-QBC targeting 40 regions on 14 mRNA transcripts was applied to K562 cells, a chronic myeloid leukemia (CML) cell line with known fusion gene, BCR-ABL^64–66^. Many of these mRNAs are known or expected to be expressed in myeloid cells^65,67–79^. K562 cells were additionally stained with a CD33 antibody (tagged with an AHCA code as in Fig. 1a) and then processed via the split-pool QBC method. Biaxial plots are shown in Fig. 9a. For CD33, we obtained single-cell expression data for protein and RNA modalities and we can compare the CD33 gene product expression levels. CD33 protein was detected in 100% of measured cells versus CD33 RNA in 85.43% of cells. There were 82.2 median protein copies per cell versus 9.43 median RNA transcripts (normalized against the other RNA targets) per cell for CD33. Nine of the 14 RNA targets (CD33, EIF2S3, PPIB, PGK1, HBE1, HBG1, GATA1, GATA2, and BCR-ABL) were detected in greater than 40% of the measured cells. The non-fusion isoforms of the ABL gene (ABLa and ABLb) were scarcely detected compared with the mutant BCR-ABL fusion transcript. We have found our normalization is skewed when normalizing across both RNA and antibody tags together due to a much higher abundance of protein copies compared with RNA transcript copies; therefore, we only normalized the RNA targets in this experiment.

To demonstrate the utility of the InsRT-QBC barcoding method for RNA analysis in heterogeneous cell populations, a primer panel to 15 RNA targets was applied to mixtures of K562 and SK-MEL-2 cell lines (Fig. 9b). SK-MEL-2 is a human melanoma cell line. There is differential expression between K562 and SK-MEL-2 cells for transcripts specific to blood cells (BCR-ABL, HBG1, HBE1, GATA1, GATA2) while expression from CD33, EIF2S3, PPIB, and PGK1 remains consistent between the mixtures. Hand gating shows that single-cell RNA expression from the BCR-ABL fusion gene transcript is correlated to the proportion of K562 cells a cell line mixture.

We found that the QBC technique, when applied to reading of point mutations, could effectively render an expression distribution for mutant alleles of a given gene. The melanoma cell line SK-MEL-2 is known to contain a SNP in the NRAS oncogene at the codon for amino acid position 61 causing an amino acid replacement from glutamine (Q) to arginine (R) (Q61R)^64^. As seen in Fig. 9c, expression of the variant allele is correlated with the proportion of SK-MEL-2 cells in the cell line mixture. The percentage of cells with mutant allele was almost double in the mixture with ~95% SK-MEL-2 cells compared with the 50:50 mixture in agreement with the nearly double number of SK-MEL-2 cells present in the cell population. Interestingly, while the majority of the cells expressed one or the other allele, there were a number of single cells that appeared to express both alleles simultaneously. Some of this co-expression may be accounted for by barcode collisions or physical cell doublets.

**Fig. 10:**
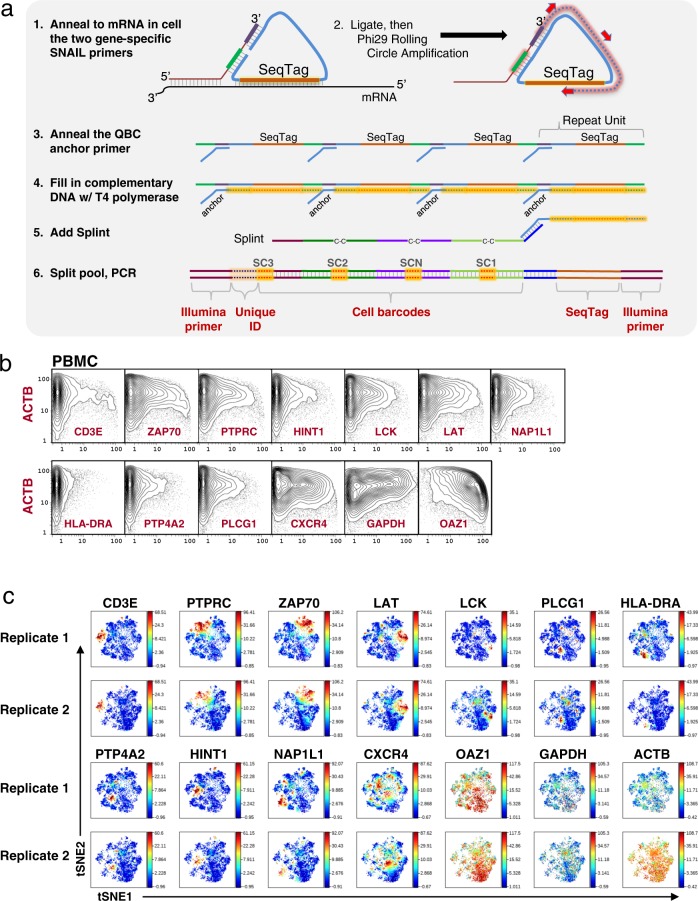
In situ amplification. **a** Schematic for target specific in situ amplification by SNAIL padlock amplification. (1) Sets of primer pairs are added to fixed cells and allowed to anneal to target mRNA. Padlock mediated ligation is performed. (2) Rolling circle amplification of the circle amplifies the sequence tag and a region complementary to the QBC anchor primer. (3) The anchor primer is added and (4) T4 polymerase extends the primer. (5) Splint is added and (6) Split-pool as per prior versions of the approach for protein and mRNA, PCR, sequencing, and data analysis. **b** Single-cell expression analysis of 14 mRNA transcripts in primary PBMC cells. Human PBMCs were prepared for SNAIL amplification against 14 mRNA transcripts. Plots represent 21,143 cells. Bi-axial plots against ACTB for all transcripts are shown. **c** Expression of T cell, B cell, and housekeeping RNA transcripts demonstrated via viSNE plots. Data from two SNAIL biological replicates were rendered via viSNE using HLA-DRA, CXCR4, HINT1, CD3E, ZAP70, LCK, LAT, PTP4A2, PTPRC, PLCG1, and NAP1L1 as selected channels to organize the viSNE plots. The housekeeping markers GAPDH, ACTB, and OAZ1 were deliberately excluded from the viSNE run to view how the expression of the other markers correlated with each other. Expression of the RNA targets varied across the cells. CD3E and HLA-DRA are known to be anti-expressed and their observed expression patterns are in non-overlapping regions of the viSNE plots.

Since cell lines are known to often express higher levels of mRNAs than primary cells, and given prior data with single-cell RNA-seq that suggests recovery from single cells of mRNA by reverse transcription is suboptimal, we investigated development of an amplification system in situ for mRNA so that the QBC process would give a higher signal for expression analysis. SNAIL-RCA was adopted for directed amplification of RNA in cells by proximity ligation. The in situ amplification workflow is shown in Fig. 10a. Two primers are used, one of which binds to the mRNA at a 3′ position and contains the sequence cognate to a common ligation junction. The second oligonucleotide contains sequence proximal, which binds slightly upstream on the mRNA, but which also anneals to the ligation junction. In this manner it is analogous to proximity ligation systems previously deployed by our group and others^12,60^, but is simpler in that it contains only two oligos requiring a single hybridization step and a single ligation event whereas prior systems rely upon four oligos, two hybridization steps, and two ligation events. SNAIL primers were designed to a panel of 14 RNA targets and applied to human PBMC primary cells (Fig. 10b, c and Supplementary Figs. 15 and 16), and human Jurkat and Nalm6 cell lines (Supplementary Fig. 17). A viSNE analysis was run on two biological replicates from human PBMC primary cells and maps of the expression profiles are shown in Fig. 10c. Expression of the markers varied across the cell populations. The replicates were consistent in that they had expression of each marker in the same area of the plots between the two replicates. There is clear expression of T cell signaling molecules such as CD3E, PTPRC, ZAP70, and LAT. There was not a high degree of co-expression between these markers suggesting that RNA expression may not correlate with protein level in these primary cells. Similar discordances were found with cytokine mRNA and protein expressions using CyTOF^12^. HLA-DRA does not co-express with CD3, as expected.

The QBC method was applied here in determination of mRNA expression levels, and expression of SNP and gene fusion mutations in hundreds of thousands of individual cells. While each sequencing library was made using approximately 50,000 cells, the number of cells labeled in each experiment was usually over 1 million, a scale not yet accessible using commercial single-cell sequencing platforms.

## Discussion

We have presented here a split-pool approach to single-cell analysis that circumvents the need to isolate single cells by building cell-specific oligo barcodes dynamically^37^. It allows barcoding of both protein and RNA in millions of single cells at once. It can surpass the parameter limits inherent to cytometry methods and (potentially) certain RNA-seq methods. The QBC method was demonstrated to detect multiple antibodies and mRNA targets in 9,000–90,000 single cells in parallel (out of barcoding mixtures of millions of cells). Up to 50 antibodies were analyzed at once, but there is no inherent limit to the number of parameters that can be assessed except for current sequencing limitations. The technique can conceivably be used to measure hundreds of mRNAs and proteins with appropriate high-throughput sequencing systems. We also demonstrated the ability to detect a BCL-ABL fusion gene transcript associated with CML and reliably detected expression of both normal and mutant alleles of an NRAS SNP implicated in melanoma. There may be challenges in scaling the targeted RNA approach due to primer interactions but massively multiplexed PCR with tens of thousands of pooled primers have been demonstrated^80,81^ and similar optimizations could be applied here. Whole transcriptome analysis could be done by replacing the reverse transcription primers with a single primer to the polyA tail and employing template switch reverse transcription or random priming to generate double-stranded products.

The cost of split-pool barcoding reagents per experiment was approximately $50. Assuming a cost of $1000 for sequencing services, the cost for barcoding and sequencing 50,000 cells is ~2.1 cents/cell. There is ~50% cell loss over 4 rounds of split-pool due to cells sticking to plasticware and supernatant removal during cell washes thus the method is better suited to barcoding large numbers of cells rather than precious samples with limited cell numbers. Pooling samples prior to split-pool can circumvent this limitation. For high throughput analysis of hundreds of samples, barcoding approaches previously developed by our group^82–85^ can be deployed. For instance, sample specific barcodes can be used (either delivered to cells via antibodies or chemical conjugation to cells) and cells pooled for staining and cell barcoding en masse with the QBC system. For example, given 1000 samples of 100,000 cells each, or 10^8^ cells, a staining of 50 antibodies could be combined in a single tube. These stained and pooled cells can be processed by QBC in a single day and sequenced on HiSeq-grade instruments (depending on the depth of the profiling required), for a nominal cost of $1.5–3 per sample (assuming a HiSeq run costs $1500) with the data returned in 1 week. To perform CyTOF at this scale would require 55 h (at 500 cells/second) or ~6 working days with associated personnel costs. Throughput and labor cost savings, plus the increased comparability across samples (samples run in one versus 6 days) is therefore attractive—especially for large clinical cohorts requiring deep profiling.

Increasing the number of targets would affect the cost of reagents minimally. The antibody reagent cost to stain 5 million cells with 50 antibody-oligo conjugates is ~$1300 and would increase as more antibodies are added. Increasing the RNA targets would similarly increase the cost for targeted primers. However, such cost considerations are the same for other single-cell analysis platforms. Whole transcriptome analysis would require fewer oligos but increase the sequencing cost to achieve the desired coverage. While antibody and sequencing costs would be similar for other single-cell assays, the cost of reagents (oligos, enzymes, buffers) needed for cell barcoding is ~$50 for 5 million cells for a 50 antibody panel, equaling fractions of a cent per cell. The power of the split-pool method is the drastically reduced cost for attaching cell-specific barcodes to targeted molecules.

As the cost of sequencing continues to drop, the cost effectiveness of using sequencing tags for a variety of purposes erstwhile occupied by fluorescence and isotope tags will come into focus. The methods presented here can be scaled to orders of magnitude more cells using additional oligo subcodes. With sample barcoding and pooling, the system may reduce batch effects inherent in other single-cell systems. Thus, QBC creates a robust, efficient, and inexpensive approach—requiring only a multi-channel pipette, four 96-well plates, enzymes and sets of oligonucleotides, to readily single-cell barcode millions of cells.

## Methods

### Antibody conjugation to a DNA oligonucleotide

Antibodies were conjugated to an amino-modified oligonucleotide according to the Solulink Antibody-Oligonucleotide All-In-One Conjugation Kit protocol. The amino-modified oligonucleotide, La4FB, was synthesized with a 5′ 4-formylbenzamide (4FB) modification and a 3′ propyl terminator modification (5′-(4FB)-AAGAGCTAGTTATTGCTCAGCGGAATAAAGCTGATGGAGTTCGTGACTGG(Propyl)-3′) (TriLink). If the provided antibody concentration was below 1 μg/ul, the antibody was concentrated first using an Amicon Ultra-0.5 Centrifugal Filter 10 K device (Millipore, UFC501024) to achieve a concentration of 1 μg/μl in 100 μl. We calculate that in successful conjugations giving satisfactory binding and subsequent sequence reads that approximately 3 oligonucleotides are conjugated to each antibody molecule. The following antibodies were obtained from Becton Dickinson: B220(RA3-6B2), CD3(17A2), CD4(RM4-4), CD41(MWReg30), CD44(IM7), CD8a(53-6.7), Ly6d(49H4), TCRb(H57-597). The following antibodies were obtained from Affymetrix: B220(RA3-6B2), CD11b(M1/70), CD19(1D3), CD3(145-2C11), CD34(RAM34), CD38(90), CD4(GK1.5), CD62L(MEL-14), CD8a(53-6.7), CD8b(H35-17.2), CXCR3(CXCR3-173), IgM(II/41), KLRG1(2F1), MHC(M5/114.15.2), PCDA1(eBio927), TCRb(H57-597), TER-119(TER-119). The following antibodies were obtained from Biolegend: CD33(WM53), CD49d(9C10(MFR4.B)), Gr1(RB6-8C5), IgM(RMM-1). The following antibodies were obtained from eBiosciences: CD135(A2F10), CD71(R17217). The following anti-human antibody oligo conjugates were obtained from Biolegend’s TotalSeq-B catalog: CD3, CD4, CD8, CD8a, TIGIT, CD274, CD27, CD28, CD25, CD137, CD19, CD20, CD24, CD45, CD45RA, CD45RO, CD56, CD69, HLA-DR, CD11b, CD14, CD11c, CD13, CD197,CD206, XCR1, CD15, CD16, CD38, CD62L, CD86, CD80, CX3CR1, CD127, CD158b, CD163, CD278, CD279, CD314, CD335, CD10, CD57, TCR α/β, TCR γ/δ, TCR Vγ9, CD155, Hashtag 1, IgG1, IgG2a, and IgG2b.

### Cell preparation

K562 cells, SK-MEL-2 cells, Jurkat cells, and Nalm6 cells were obtained from America Type Tissue Collection (ATCC). K562 cells were grown in IMDM media (from Invitrogen) with 10% FBS and 1% Pen Strep Glut. SK-MEL-2 were grown in EMEM (from ATCC) with 10% FBA and 1% PenStrep Glut. Spleen cells were obtained from C57/Blk6 mice, between 6 and 8 weeks of age in compliance with Stanford IACUC ethics regulations. PBMCs were obtained from donors though the Stanford Hospital. 16% formaldehyde solution (Thermo Scientific, 28908) was added directly to cells to a final concentration of 1.6% and incubated for 10 min at room temperature under gentle agitation to fix the cells. Cells to be analyzed for only protein expression were stored in 1X PBS with 10% DMSO at −80 °C until antibody staining. Cells to be analyzed for only mRNA expression or both protein and mRNA expression were pelleted and permeabilized with ice-cold methanol for at least 10 min on ice. Once in methanol, cells were stored at −80 °C until processing for QBC.

### In-cell reverse transcription

Cells stored in methanol were washed two times with 1X PBST (1X PBS, 0.1% Tween 20) containing 40 U of RNAsin Plus RNase Inhibitor (Promega, N2615) per ml of 1X PBST. All cell washes in all steps were carried out with 1 ml of wash solution followed by centrifuging at 600×*g* for 3 min per wash and aspirating the supernatant. The cells were treated with a mild proteinase K digestion to make them permeable to the reverse transcriptase enzyme and slightly degrade RNA binding proteins that are known to inhibit reverse transcription. The optimal conditions for pretreatment of cells with proteinase K varied depending upon cell type with final concentration of proteinase K (NEB, P8107S) between 0.01 and 0.02 μg/ml and incubation at 37 °C for between 5 and 10 min. Cells were again washed two times with 1X PBST containing RNase inhibitor. Cells were divided into reaction tubes containing ~8 million cells per tube and re-suspended in 250 μl reverse transcription reactions: 1.5–2 μM of each reverse transcription primer (Supplementary Data 5–7), 1 mg/ml Salmon Sperm DNA (Invitrogen, AM9680), 0.25 mM dNTP Solution Mix (NEB, N0447), 0.25 mM Aminoallyl dUTP (Thermo Scientific, R0091), 5000 units ProtoScript II Reverse Transcriptase (NEB, M0368), 1X ProtoScript II reaction buffer (NEB, B0368), 500 units of SUPERase-In RNase Inhibitor (Ambion, AM2696), and 10 mM DTT in 1X PBS. The reverse transcription reactions were heated to 65 °C for 3 min and placed on ice before adding the reverse transcriptase enzyme and the RNase inhibitor enzyme followed by incubation at 42 °C for between 30 min and 1 h.

### Crosslinking of aminoallyl nucleotides in cDNA

The cells were washed two times with 1 ml of cold 1X PBS. Cells were re-suspended in 1 ml of 1X PBS containing 2 mM DTSSP (Thermo Scientific, 21578), an amine-reactive linker, and incubated for 30 min at room temperature. To stop the crosslinking reaction, Tris, pH 7.5 was added to the cell suspension to a final concentration of 100 mM and incubated for 15 min at room temperature. Cells intended for protein expression analysis in addition to mRNA expression analysis were stained with oligonucleotide-conjugated antibodies as described next. If cells were only to be analyzed for mRNA expression, they were washed two times with HSM (1x PBS, 0.5% BSA, 0.02% Sodium Azide, 0.5 M NaCl) followed by quantum barcoding by split-pool synthesis described later.

### Cell staining with oligonucleotide-conjugated antibodies

Thawed cells stored in 1X PBS with 10% DMSO or cells with crosslinked cDNA that are also to be analyzed for proteins were washed three times with SME (1x PBS, 0.5% BSA, 0.02% Sodium Azide, 5 mM EDTA). All cell washes in all steps were done with 1 ml of wash solution followed by centrifuging at 600 × g for 3 min per wash and aspirating the supernatant. Cells were incubated for 30 min at room temperature with shaking in 200 μl of blocking buffer. Blocking buffer for mouse cells contained 0.5 M NaCl, 0.285 mg/ml ChromPure Mouse IgG (Jackson ImmunoResearch, 015–000–003), and 0.2 mg/ml Salmon Sperm DNA (Sigma-Aldrich, D7656), in SME buffer. Blocking buffer for human cells contained 0.5 M NaCl, 4–50 μl Human TruStain FcX blocking solution (BioLegend, 422302), and 0.2 mg/ml Salmon Sperm DNA (Sigma, D7656) in SME buffer. Each antibody-La4FB conjugate was incubated with a different AHCA oligonucleotide (5′-(phos)-CTCCCTGTCTGACG(xxxxxxxxx)AGATCGGAAGAGCACACGTCTGAACTCCAGTCACGAACTCCATCAGC-3′)(IDT) (where x = AHCA code per Supplementary Data 1 and 2) at equal molar concentration as the La4FB oligo for 1 h at 37 °C with rotation. We used approximately 0.2 μg of each antibody per 100 μl of total staining volume. Some antibodies were titrated up or down depending on the antibody pool, binding affinity and specificity. When staining with multiple antibody-La4FB conjugates, each antibody-La4FB conjugate was hybridized to a different AHCA oligo. Following AHCA hybridization, the antibody conjugates were combined and added directly to the cells in blocking buffer. NaCl was added to bring the final salt concentration to 0.5–0.65 M. A typical total staining volume was approximately 300 μl. Cells were stained for 2–3 h at room temperature with rotation. After staining, cells were washed three times with HSME (SME containing 0.5 M NaCl). Following the final wash, cells were re-suspended in 1.2 ml of fixing solution containing 4% formaldehyde diluted in HSME at room temperature for 10 min (murine cell staining) or 30 min (human cell staining) with rotation. For murine cell antibody staining experiments, cells were centrifuged again, re-suspended in a second formaldehyde fixing reaction with the same conditions as the first fixing except incubated at room temperature for 20 min. Cells were washed two times with HSM.

### Quantum barcoding by split-pool synthesis

After the final wash with HSM, cells were re-suspended in a 200 μl Splint6 hybridization reaction. For cells stained with antibody-oligo conjugates, a 3:1 molar ratio of Splint6 oligonucleotide (5′-CGTCAGACAGGGAGCGGTGGTTGG/iSpC3/GGAAGTCGCCCACAACGG/iSpC3/GGCAAAGACGAGAATGGG-/iSpC3/GGGTAACGCACTAGGACTTGGTGAGC/3SpC3/-3′) (synthesized by IDT) to the antibody-conjugated oligonucleotide amount added during staining was used. For cells that were only analyzed for mRNA expression, a final concentration of 0.1–0.5 μM of Splint6 oligonucleotide was used. The reaction was incubated at 37 °C for 1 h with rotation. For human cells stained with TotalSeqB antibodies, prior to Splint6 hybridization, cells were washed with 1 ml of HSM, incubated with between 1 and 3 μM TotalSeq-B linker oligo (5′-/5Phos/GCTCCCTGTCTGACGTTGCTAGGACCGGCCTTAAAGC-3′) (synthesized by IDT) in 200 μl at 37 °C for 1 h with rotation followed by another wash with 1 ml of HSM. After Splint6 hybridization, cells were dyed in 0.07% Trypan Blue Dye (Bio-Rad, 1450013) for 5 min at room temperature to facilitate visualization of cell pellets during split-pool. Cells were washed once with 1 ml of HSM and re-suspended in an appropriate volume of ligation mix calculated for each split. Cells underwent four rounds of split-pool with each round consisting of the following steps: cells were divided into 60 wells, a different subcode was added to each well (Supplementary Data 3 and 4), subcode oligonucleotides were annealed to the Splint6 sequence, annealed subcodes were ligated to cDNA or linker sequence, divided cells were washed with 100 μl of HSM and centrifuged to remove excess subcode oligonucleotides, the 60 wells were pooled into a single tube, cells were washed with 1 ml of HSM, re-suspended in the calculated volume of ligation mix necessary for splitting into 60 wells, and split again to repeat the process.

Subcode annealing and ligation was done simultaneously in 20 μl reactions per well containing between 0.1–2 μM of subcode oligonucleotide keeping a 1:3 molar ratio of Splint6 oligo to subcode oligos, 1X PRL buffer (65 mM Tris-HCl, PH7.5, 10 mM MgCl_2_, 8% PEG 3350), 0.3 mM ATP (NEB, P0756), and 80 units T4 DNA Ligase (NEB, M0202). The plate of 60 reactions was incubated at room temperature for 15 min with shaking.

After the final pooling, cells were washed once with HSM, re-suspended in water, and the cell concentration was measured by a TC20 Automated Cell Counter (Bio-Rad).

### Post-split-pool library preparation for cDNA generated by InsRT-QBC

Following split-pool, RNA was degraded and cells were lysed to make cDNA accessible for PCR and to denature proteins that inhibit PCR. 50,000 cells were pipetted into a 0.2 ml tube containing 5 U of RNase H (NEB, M0297), 1X RNase H reaction buffer (NEB, B0297), and 10 mM DTT in a total volume of 20 μl. The reaction was incubated at 37 °C for 30 min. Proteinase K (NEB, P8107S) was added to the cells to a final concentration of 1.4 mg/ml and incubated at 55 °C for 1 h to lyse the cells. The reaction tube was heated to 95 C for 10 min to inactivate the Proteinase K. A mix containing components for second strand cDNA synthesis was added directly to the reaction tube to bring the volume to 50 μl with the following final concentrations of mix components: 0.4 mM of each dNTP (Sigma, D7295), 0.025 μM of each RNA-specific PCR primer (Supplementary Data 8–10), 0.5 μl Herculase II Fusion DNA Polymerase (Agilent, 600675), and 1X Herculase II Reaction Buffer (Agilent, 600675). Thermocycling was done with an initial denaturation at 95 °C for 2 min followed by between 1 and 20 cycles of denaturation at 95 °C for 20 s, annealing at 62 °C for 20 s, and extension at 68 °C for 1 min, and a final extension at 68 °C for 4 min. Following second strand synthesis, aliquots from mixtures of mouse cell line BW5147 and human cell line Nalm6 and mixtures of human Jurkat and Nalm6 cell lines were additionally incubated with 1.5 μl of Thermolabile Exonuclease I (NEB) for 6 min at 37 °C followed by inactivation at 80 °C for 1 min before proceeding to PCR amplification. Exonuclease treatment was intended to degrade any remaining single-stranded unligated subcode oligos in the reactions that could act as primers during the PCR reaction and contribute to noise in the resulting sequencing data.

### PCR amplification

After second strand synthesis of cDNA from cells analyzed for mRNA expression, additional components necessary for PCR were added directly to the reaction tube for a final volume of 53 μl and the following final concentrations of additional components: 0.25 mM of each dNTP (Sigma, D7295), 0.25 μM each of NEBNext Illumina forward and reverse index primers (NEB, E7600S), and 1 μl Herculase II Fusion DNA Polymerase (Agilent, 600675). Thermocycling was done as in second strand synthesis but with an annealing temperature of 67 °C and between 12 and 30 cycles.

For cells that were analyzed for only protein expression, 50,000 cells were added to a PCR tube and PCR mix was added directly to cells. A final PCR volume of 50 μl contained 0.2 mM each dNTP (NEB, N0447), 0.2 μM each of NEBNext Illumina forward and reverse index primers (NEB, E7335), 1x Pfu Ultra II reaction buffer (Agilent, 600672), and 1 μl PfuUltra II Fusion HS DNA Polymerase (Agilent, 600672). Thermocycling was done with an initial denaturation at 94 °C for 3 min followed by 24–28 cycles of denaturation at 94 °C for 25 s, annealing at 65 °C for 30 s, and extension at 70 °C for 1 min, and a final extension at 70 °C for 3 min.

For human PBMCs that were stained with TotalSeq-B (BioLegend) antibodies, prior to PCR, a polymerase extension step was done to copy the epitope tag sequence to the same strand as the subcodes. Following split-pool, approximately 500,000 PBMCs were resuspended in a polymerase extension solution containing 0.24 mM of each dNTP (NEB, N0447), 1x Phi29 Polymerase Buffer (NEB, M0269), 0.1 mg/ml BSA (NEB, B9000S), and 30 U of Phi29 Polymerase (NEB, M0269) in a total volume of 500 μl. The reaction was incubated at 30 °C for 30 min followed by one wash in HSM buffer. These cells were re-suspended in water and the cell concentration was measured by a TC20 Automated Cell Counter (Bio-Rad). In all, 50,000 cells were aliquoted into PCR tubes and PCR was carried out as described in the protein-only PCR protocol above.

PCR products were visualized on either 2% agarose gel by electrophoresis or by Bioanalyzer DNA 1000 kit. DNA fragments of the expected sizes were either cut from the gel and purified using the QIAquick Gel Extraction Kit (Qiagen, 28704) or DNA fragments were cleaned up by bead purification with KAPA Pure Beads (Roche, 07983298001). Samples purified by gel purification and bead purification produced comparable sequencing data.

### Primer design

RNA specific reverse transcription and second strand synthesis primer pairs were designed using Primer3 software (http://bioinfo.ut.ee/primer3/). Primer selection was done either manually or using Roche proprietary primer filtering software which takes the following metrics into consideration: hybridization temperatures and ∆G of primer annealing, oligo and binding site secondary structure, amplicon length, repetitive regions and common SNPs in target sequence, RNA template and DNA amplicon secondary structure, off-target transcriptome products, and multiplexing primer-primer/primer-product interactions. Primer sequences were checked for sequence specificity using Blat software (http://genome.ucsc.edu/cgi-bin/hgBlat). The primer sets targeted mRNA sequence regions with lengths between 40 and 134 bp. The primer lengths were 22 bp ± 6 bp. The GC% was between 20 and 80. RT primers were designed with Tm of 49 °C ± 6 °C or 65 °C ± 6 °C. The Splint annealing anchor sequence (5′GCTCCCTGTCTGACG) was synthesized on the 5′ end of the reverse transcription primers and the 5′ ends were modified to terminate in a phosphate. The RNA specific 2nd strand synthesis primers were designed with Tm of 62 °C ± 6 °C. They were synthesized with the Illumina primer annealing sequence (5′GTGACTGGAGTTCAGACGTGTGCTCTTCCGATCT) on the 5′ end. In mouse-human species mixing experiments, due to sequence homology, primers designed to the mouse transcriptome were observed to initiate reverse transcription on human transcripts and vice versa. The targeted primer pool was refined to primers upstream of sequence with enough sequence divergence that species type could be effectively determined. Furthermore, perfect sequence match was enforced during data analysis to reduce species crosstalk.

### SNAIL probe design

Between 2 and 5 SNAIL probe pairs were designed for each RNA target (Supplementary Data 11). The probe pairs were distributed along the transcripts because we previously observed variation in probe efficiency likely due to the presence of RNA secondary structures and RNA binding proteins. SNAIL probes were designed at a distance of 0–8 nucleotides from each other, using Primer3plus, with the following settings: Primer length: Min. = 18 Opt. = 20 Max. = 27, Primer Tm: Min. = 57 Opt. = 60 Max. = 63, Primer GC%: Min. = 20 Max. = 80, Product size range: 30–60.

### SNAIL-probe hybridization

SNAIL probes were resuspended in DEPC-treated water at a concentration of 100 μM. SNAIL-padlock and SNAIL-splint probes for all target transcripts of an experiment were mixed and heated to 90 °C for 5 min. Probes were then chilled on ice and added to cells in hybridization buffer at a final concentration of 100 nM. Paraformaldehyde-fixed and methanol-permeabilized cells (see above) were pelleted by centrifugation at 600 g for 3 min. Cells were washed once with with 1X PBST containing RNase inhibitor (see above). Hybridization with SNAIL probes was performed in a buffer based on DEPC-treated water (Life Technologies) containing 1× SSC (Affymetrix), 2.5 % v/v polyvinylsulfonic acid, 20 mM ribonucleoside vanadyl complex (New England Biolabs), 40 U/mL RNasin, 1% Tween, and 100 μg/mL salmon sperm DNA (Life Technologies). Cells were incubated for 1 h at 40 °C under vigorous agitation, and subsequently washed three times with 1X PBST containing RNase inhibitor.

### SNAIL high-salt wash

Cells were then incubated for 20 min in a buffer containing PBS, 4× SSC, 40 U/mL RNasin at 40 °C under vigorous agitation.

### SNAIL ligation

After two washes with 1X PBST containing RNase inhibitor, cells were incubated for 1.5 h with T4 DNA ligase (Thermo Scientific, EL0012) at room temperature with gentle agitation and following manufacturers’ instruction, with addition of 40 U/mL RNasin.

### SNAIL amplification

After two washes with 1X PBST containing RNase inhibitor, cells were incubated for 3 h with phi29 DNA polymerase (Thermo Scientific, EP0091) at 30 °C, under agitation and following manufacturers’ instruction, with addition of 40 U/mL RNasin. Longer amplification (up to 16 h) generally increases signal intensity.

### SNAIL QBC-anchor hybridization

Cells were incubated with SNAIL-QBC-anchor oligonucleotide (5′-pGCTCCCTGTCTGACGCATACACTAAAGATAACAT) at a concentration of 5 nM for 8 h at 37 °C in PBS, 1× SSC, 0.1% Tween, 40 U/mL RNasin. The annealed SNAIL-QBC-anchor oligonucleotide was extended by T4 DNA polymerase to synthesize the sequence complementary to the RNA SeqTag. Cells were washed once with 1X PBST and resuspended in a reaction containing 0.2 μM QBC-anchor oligonucleotide, 0.1 mg/ml BSA, 0.1 mM of each dNTP (NEB, N0447), 1x PRL buffer, and 3 units T4 DNA polymerase (NEB, M0203S) in 1xPBS to total volume of 100 μl. The reaction was incubated at 37 °C for 30 min. Cells were washed two times with HSM buffer. Splint added and split-pool as described above

### Post-split-pool library preparation for SNAIL samples

Cells were lysed in 1.4 mg/ml Proteinase K at 55 °C for 1 h and heated to 95 °C for 10 min to inactivate the Proteinase K. The SNAIL Illumina primer (5′-GTGACTGGAGTTCAGACGTGTGCTCTTCCGATCTTTTCAGTAATAATT) was heated at 95 °C for 5 min and added to a final concentration of 0.025 uM in 1X Phi29 reaction buffer (NEB, M0269S) to the lysis product and allowed to anneal for 15 min at 37 °C followed by 15 min at room temperature. After annealing the SNAIL Illumina primer, 0.25 mM of each dNTP (NEB, N0447), 4 mM DTT, and 5 units of Phi29 DNA polymerase (NEB, M0269S) were added in a final volume of 25 μl and incubated at 30 °C for 1 h. The enzyme was inactivated at 65 °C for 10 min. PCR was performed as described above for Ins-RT-QBC.

### DNA sequencing and analysis

Purified PCR products were sequenced by MiSeq, HiSeq, and NextSeq (Illumina) instruments for between 151 and 258 bp single-end or paired-end reads. FLASH (https://ccb.jhu.edu/software/FLASH/ version FLASH-1.2.11) was used to overlap paired-end reads. The fasta files were processed with a software package written to de-convolute barcode sequences into single cells. An expanded discussion of the file processing is provided in the online materials, including the code files for processing.

### Standard preparation of reads and pre-alignment filtering

De-multiplexed FASTQs were obtained after sequencing by using the Illumina-provided Bcl2Fastq software package v2.18. Paired-end reads are merged (FLASH-1.2.11) and converted to fasta (seqtk version 1.2).

### Parsing, deduplicating and filtering—QBC-parse_v1.0

Deduplication based on unique molecular identifiers (UMI)—were performed using the QBC-parse_v1.0, which allows alignment of sequences with one mismatch. For RNA data, we found that samples treated with exonuclease to remove remaining single stranded subcode sequences before PCR amplification, had higher read duplication rate. For these samples, we required UMIs to be supported by at least two reads. Removing singleton reads (reads sequenced only once) can reduce noise derived from amplification artifacts. The QBC algorithm sequentially: (a) detects barcode via alignments, (b) corrects barcode by efficiently comparing barcodes to a whitelist, (c) deduplicates based on UMI, (d) evaluates reads for chimera filtering (check for evidence of PCR-based cross-over), (e) filters reads for underrepresented/artificially created cells, and (f) transforms sequences into table of cells and markers. Executable and script to process fasta to FCS can be found at https://github.com/bioinform/QBC_Single_Cell_Analysis_NGS.

### Filtering of cells after QBC-parse

For each cell we quantified the number of markers with at least one unique mapped read, and then excluded: cells with too few counts of expressed markers, cells with too few positive markers and cells with too many positive markers. Following quality control, expression values Ei,j for marker i in cell j were normalized by dividing the unique read counts for marker i by the mean of the marker counts in cell j and multiplying by 10, to account for differences in coverage per cell (arithmetic mean). This matrix with normalized expression counts is then used as input for further downstream analysis. We have performed normalization in all samples this way except for experiments in which the marker panel consisted of both RNA and protein targets. Experiments wherein the target panel consisted of both RNA and protein, the expression counts for protein were much higher than RNA targets which can be attributed to the difference in detection modalities between the two. In such cases, we retained the raw expression counts for protein markers and normalized the expression counts of RNA targets alone.

### Dimensionality reduction using X-shift, t-SNE and viSNE

High quality cells with normalized marker count values are jittered by the standard python matplotlib to avoid overlapping datapoints in a scatter plot for visual inspection. Normalized data is input into X-shift (v.14.Mar.2018). Eventnum and housekeeping genes are excluded from clustering with no additional rescaling. Clustering was performed with Euclidean distance measurement and “elbow point” is empirically calculated for each set of sequencing runs to determine the optimal number of clusters. The resulting data is used to create Minimum Spanning Trees within the Vortex analysis pipeline^50^.

For tSNE^63^ plots, we used Rtsne package in R to map the high dimensional normalized expression matrix, comprising of 29 features, to a 2-dimensional sub-space. We used all RNA targets as clustering parameters. We set the learning rate to 1000, perplexity to 30 while running the Rtsne function. Cluster assignment of cells (i.e., color coding) was defined by assigning cells to Human or Mouse if ≥75% of the expressed features belong to a particular species and Collision if neither.

We used normalized data input into Cytobank^48^ to generate viSNE^57^ visualizations. We excluded housekeeping or stably expressed targets, Hashtag antibody, or isotype controls from the clustering analyses. Number of iterations was set to 1000, Perplexity was set to between 30 and 100, and Theta was set to 0.5.

### Statistics and reproducibility

Biological replicates were performed on different samples of the same cell types (cell line, primary mouse cells or primary human PBMC cells) on different days and/or by different operators. Three samples had two biological replicates and one sample had three biological replicates. Multiple aliquots of split-pool barcoded cells from the same mouse and human cell line mixture experiment were separately prepared with a library preparation PCR and sequenced. These aliquots gave similar results as seen in Supplementary Fig. 10.

### Reporting summary

Further information on research design is available in the Nature Research Reporting Summary linked to this article.

## Supplementary information

Supplementary Information

Description of Additional Supplementary Items

Supplementary Data

Reporting Summary

## Data Availability

Raw sequencing data, processed data in the form of normalized and unnormalized text files, and files needed to process raw data have been deposited into the Gene Expression Omnibus with accession number GSE130784.

## Code availability

Executable and script to process fastq to FCS can be found here:

https://github.com/bioinform/QBC_Single_Cell_Analysis_NGS. It has also been deposited into a DOI-minting repository at https://zenodo.org/record/3634978 ^86^.
