# Author Correction: Ultra-high throughput single-cell analysis of proteins and RNAs by split-pool synthesis

**DOI:** 10.1038/s42003-020-1019-9

**Published:** 2020-05-29

**Authors:** Maeve O’Huallachain, Felice-Alessio Bava, Mary Shen, Carolina Dallett, Sri Paladugu, Nikolay Samusik, Simon Yu, Razika Hussein, Grantland R. Hillman, Samuel Higgins, Melanie Lou, Angelica Trejo, Laura Qin, Yu Chuan Tai, Shigemi M. Kinoshita, Astraea Jager, Deval Lashkari, Yury Goltsev, Sedide Ozturk, Garry P. Nolan

**Affiliations:** 1 Roche Sequencing Solutions, Pleasanton, CA USA; 2 Apprise Biosciences, Menlo Park, CA USA; 3 Department of Microbiology and Immunology, Baxter Laboratory for Stem Cell Research, Stanford University School of Medicine, Stanford, CA USA

**Keywords:** Immunogenetics, Diagnostic markers, Proteomic analysis, Tumour immunology, Transcriptomics

Correction to: *Communications Biology* 10.1038/s42003-020-0896-2, published online 7 May 2020.

In the original published version of the article, the name of co-author Samuel Higgins was spelled incorrectly. In addition, there were minor errors in Figures 2, 3, 4, 7, and 8 that affected the clarity of the presented information. The errors have been corrected in the HTML and PDF versions of the paper.

