# Supplementary material for: Ultra-high throughput single-cell analysis of proteins and RNAs by split-pool synthesis
Source: Commun Biol. 2020 May 7;3:213. doi: 10.1038/s42003-020-0896-2 (PMC7205613; doi:10.1038/s42003-020-0896-2)
Supplement: Supplementary file 1 — Supplementary Information [file 42003_2020_896_MOESM1_ESM.pdf]

Supplementary Figure 1

a

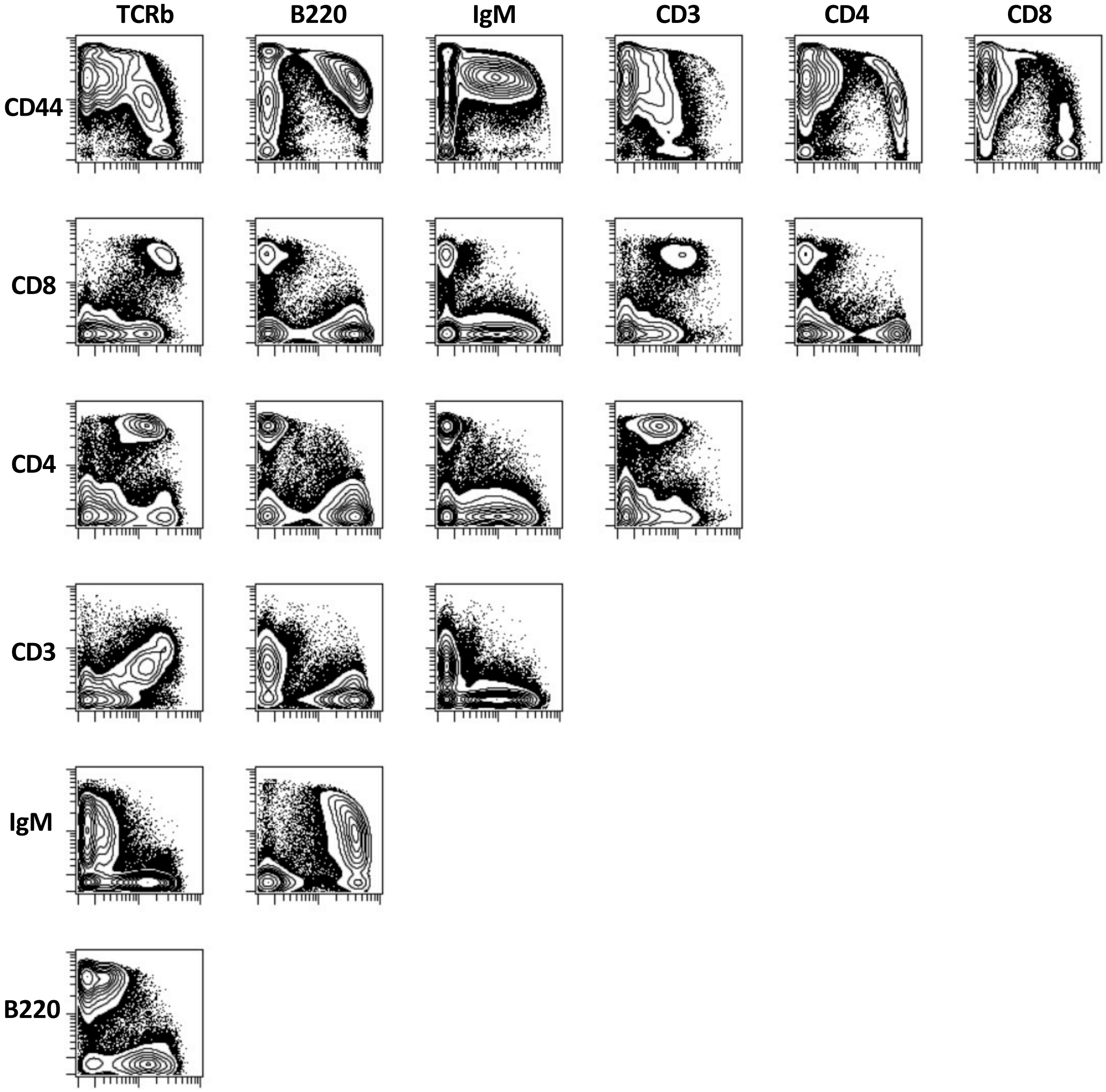

b

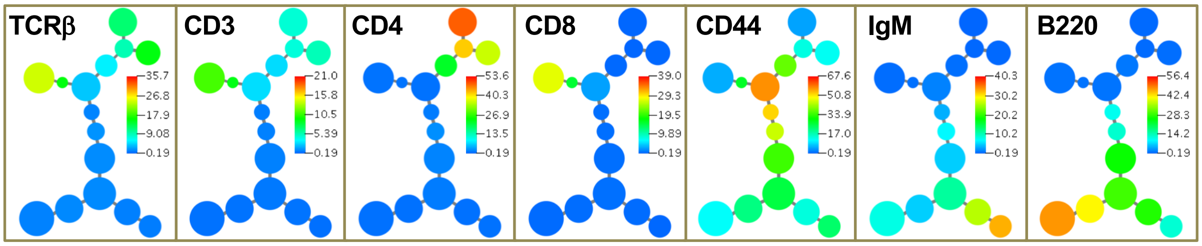

a. Pairwise biaxial plots for data in Figure 2A. Wildtype murine spleen were stained with 7 different antibodies, each barcoded with a unique epitope code (AHCA) per Figure 1A. Antibodies used were directed against epitopes on TCRb, CD3, CD4, CD8, CD44, B220, and IgM. 63,870 cells were detected by the analysis and represented in the plots. Antibodies used are shown on the axes.

b. Clustering and MST representation of stained splenic cells, from Supplementary Figure 1A, above. An X-shift driven minimum spanning tree is shown for the FCS data for the markers used in Figure 2a. The identical MST to that in main Figures 2b is provided for convenience and comparison to the biaxial plots above. All 7 markers were used in the clustering run.

Supplementary Figure 2

a

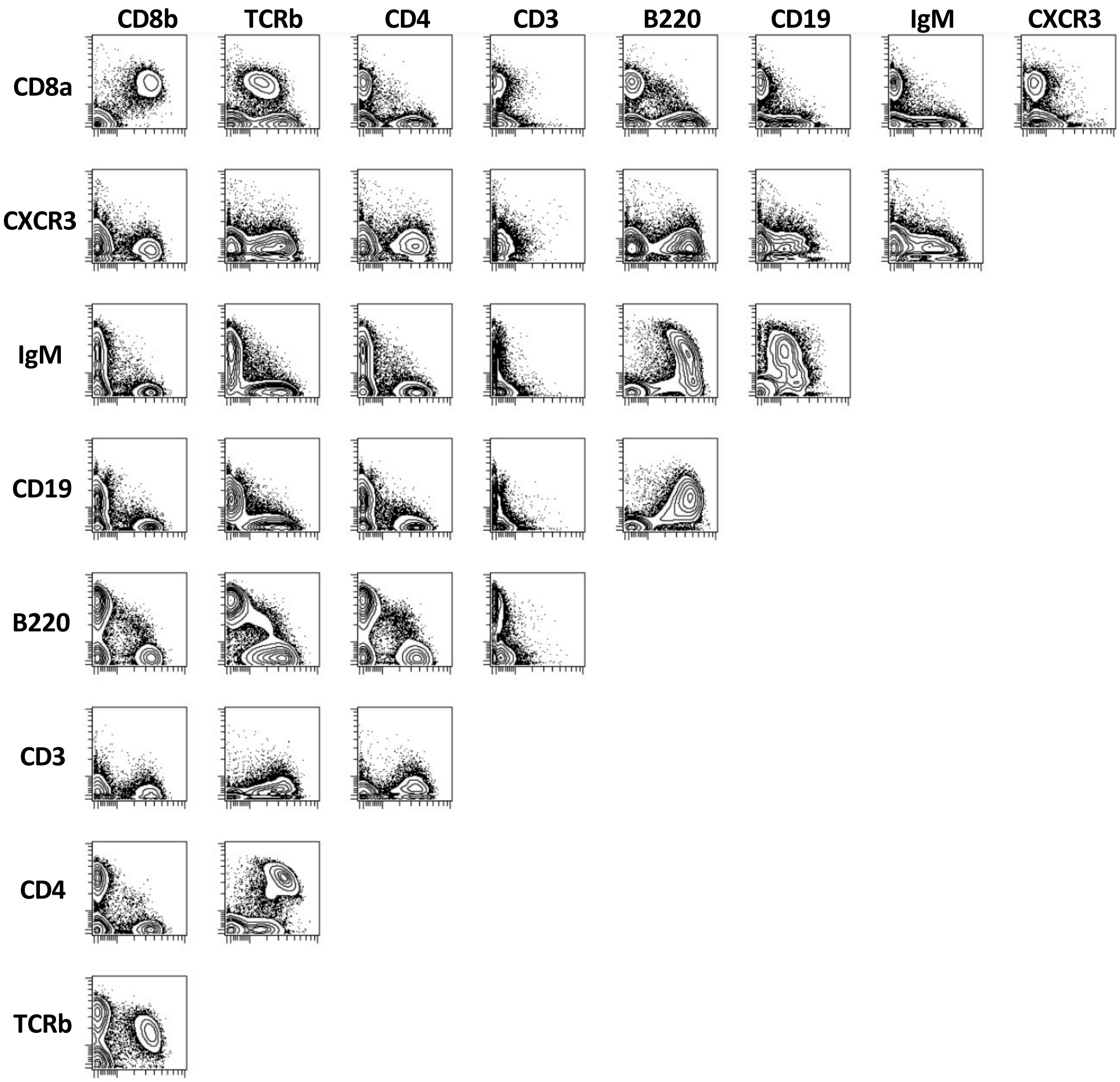

b

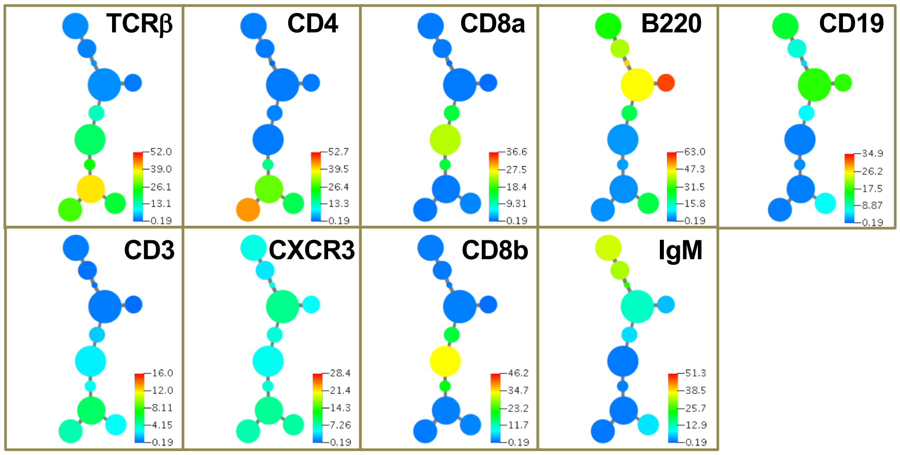

Supplementary Figure 2

a. Pairwise biaxial plots for normalized data in Figure 2c**.** Multiple antibodies (CD8a, CD8b, TCRb, CD4, CD3, B220, CD19, IgM, CXCR3) were used to stain murine splenic cells. Antibodies were used that are specific to two distinct epitopes present on the heterodimeric chains of CD8 (designated CD8a and CD8b). Expression data read from sequences is shown in the figure, with the expected correlation observed. 21,307 cells represented.

b. Clustering and MST representation of stained splenic cells, from Supplementary Figure 2A, above. An X-shift driven minimum spanning tree is shown for the .fcs data for the markers used in Supplementary Figure 2a. The identical MST to that in main Figures 2d for convenience and comparison to the biaxial plots above. All 9 markers were used in the clustering run.

Supplementary Figure 3

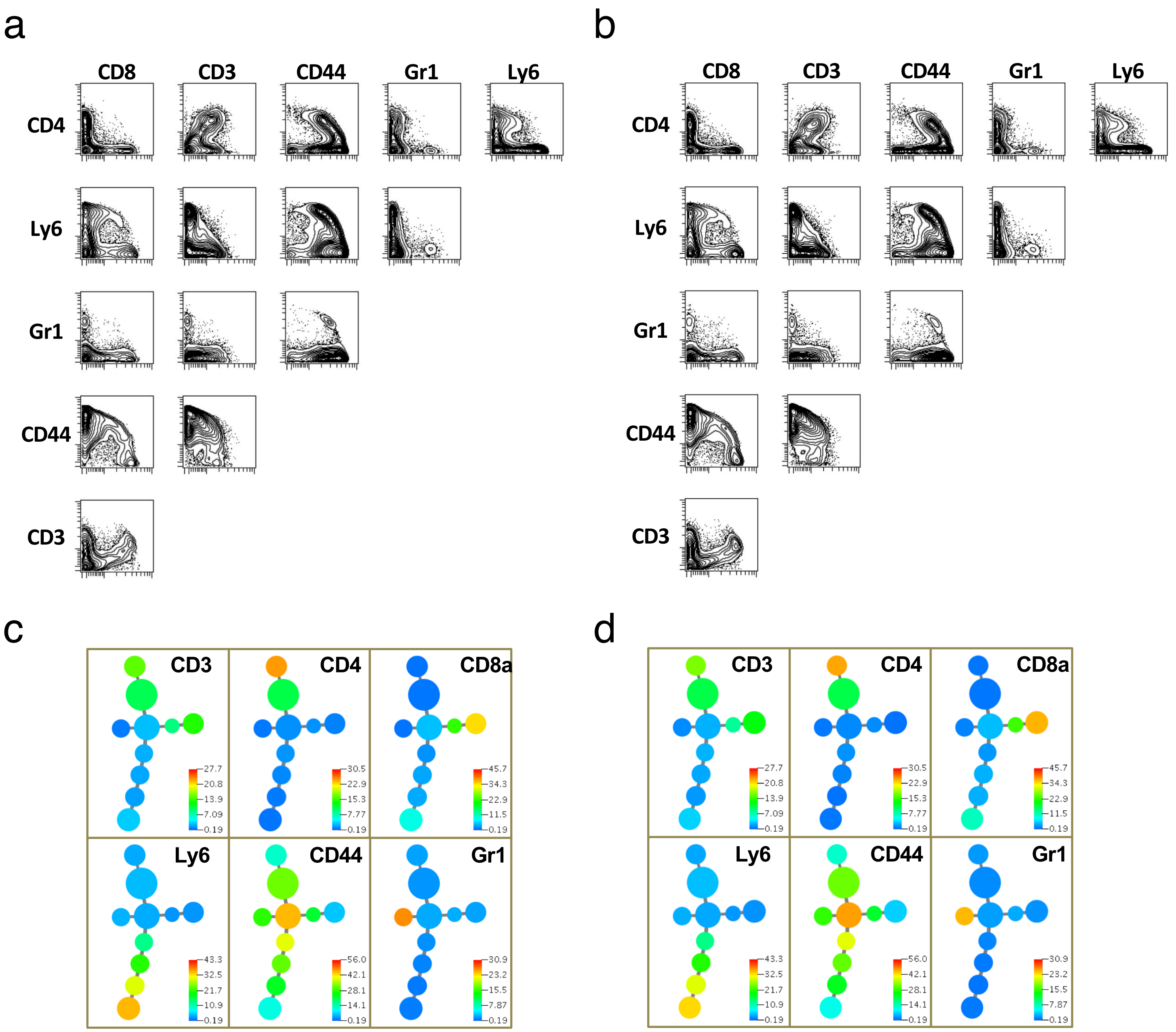

a. Pairwise biaxial plots for data in Figure 3a replicate 1.

b. Pairwise biaxial plots for data in Figure 3a replicate 2.

c. Clustering and MST representation of stained splenic cells for data from Supplementary Figure 3a, above.

d. Clustering and MST representation of stained splenic cells for data from Supplementary Figure 3b, above.

Supplementary Figure 4

b

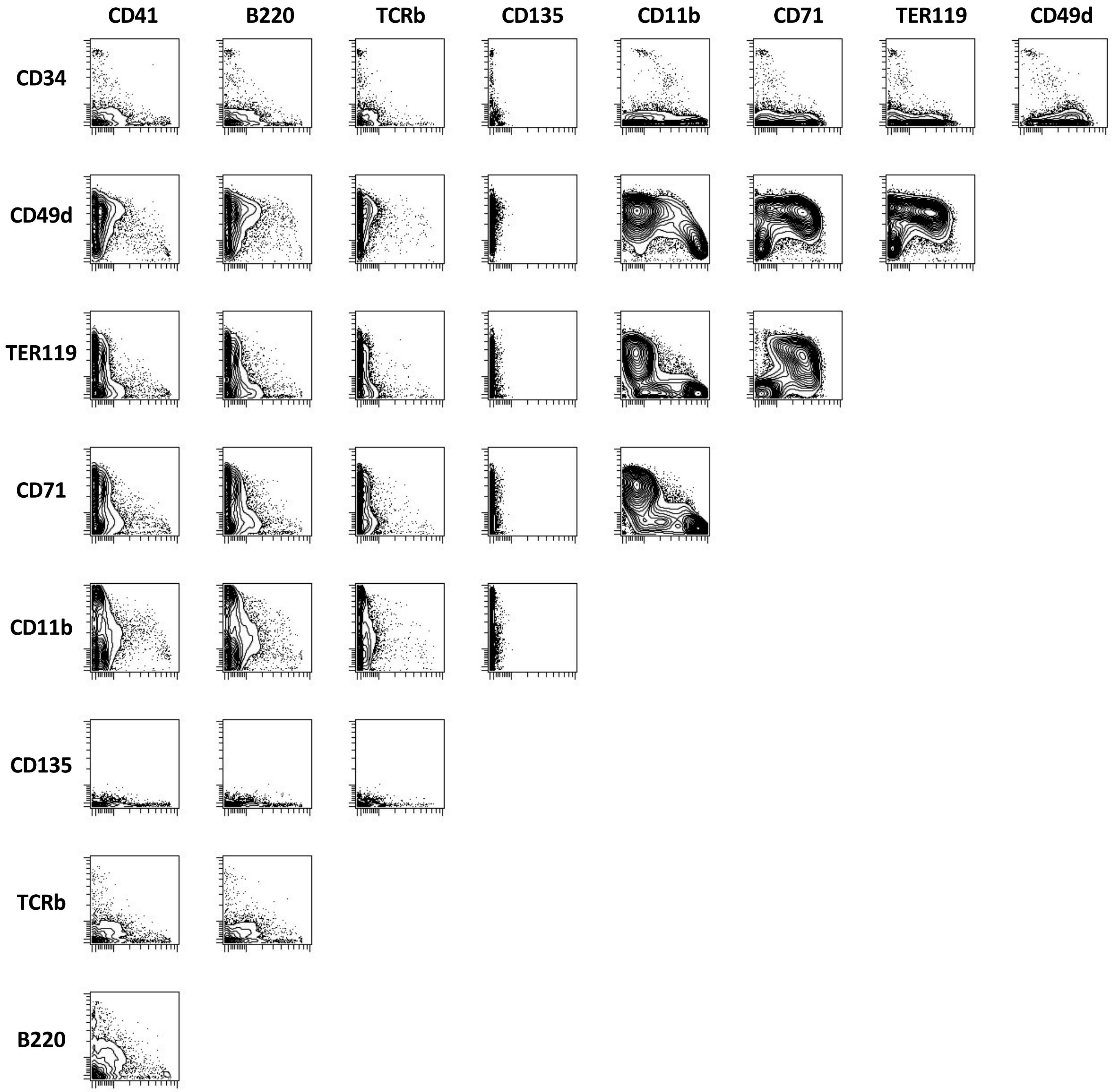

Pairwise biaxial plots for data in Figure 3c. 12,373 bone marrow cells were detected with oligo-conjugated antibodies specific for TCRb, B220, CD11b, CD34, CD49d, CD71, TER119, CD135, and CD41.

Supplementary Figure 5

a

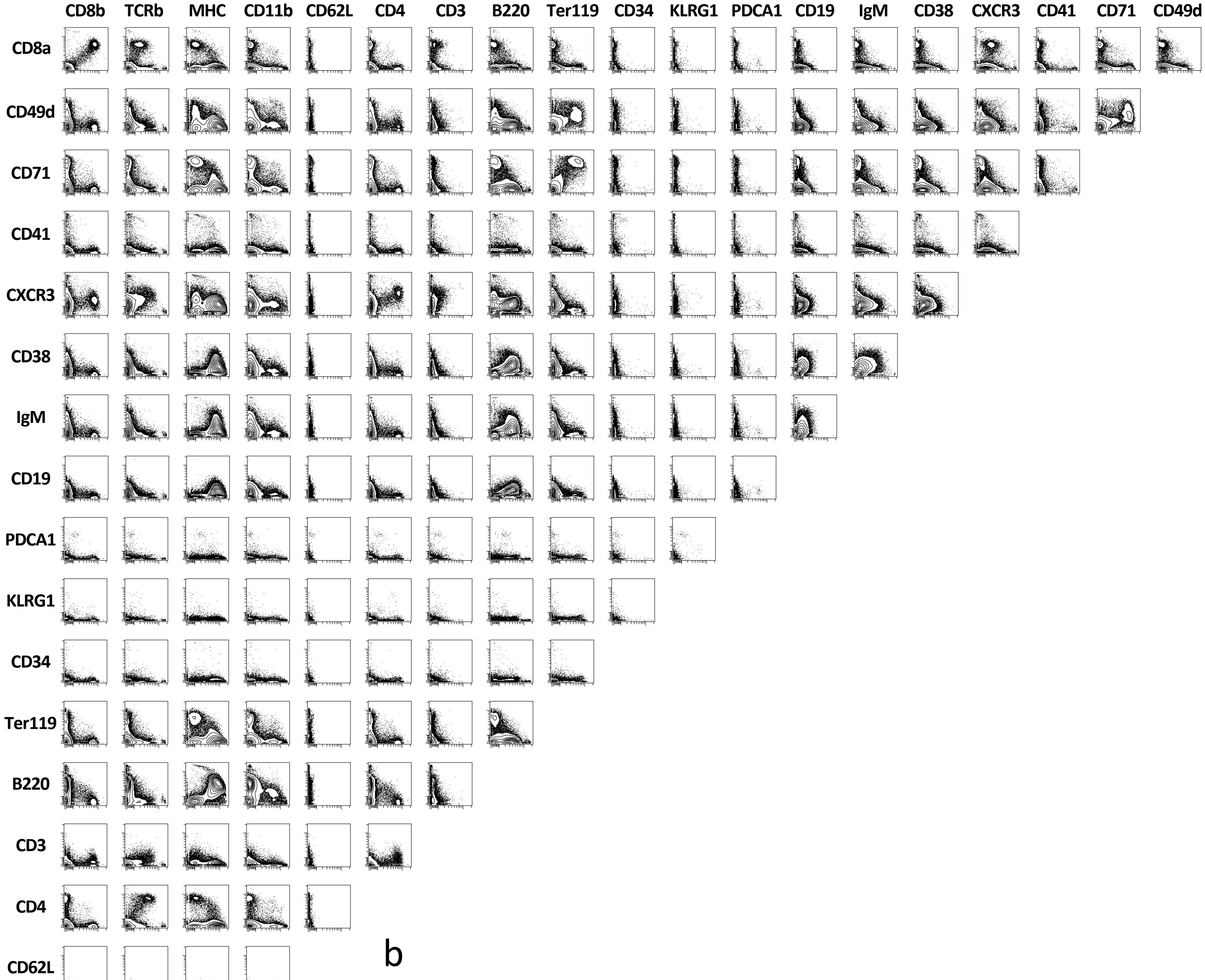
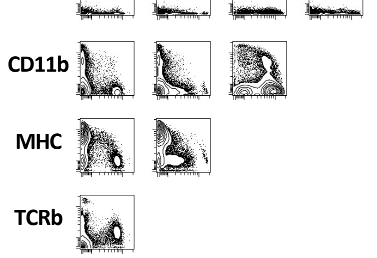

b

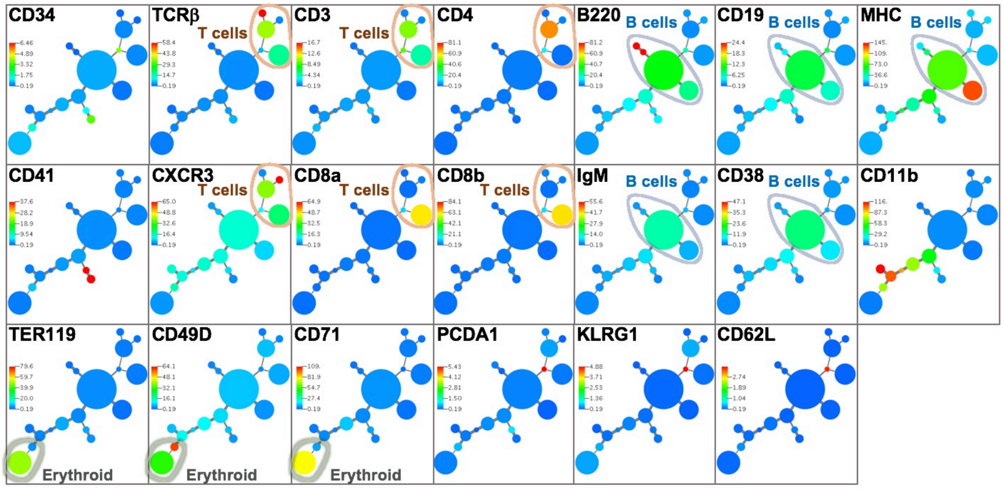

Supplementary Figure 5

a. Pairwise biaxial plots for data in Figure 4a**.** A 50:50 mixture of splenic and bone marrow cells was analyzed with QBC and the analysis outcome representing 34,150 cells plotted. Expression profiles and percentages match cell expression profiles and cell percentages expected for these murine organs. 20 antibodies were used in this staining: TCRb, CD3, CD4, CD8a, CD8b, MHC, CD62L, B220, IgM, CD19, CD38, CD11b, CXCR3, CD34, PCDA1, CD49d, CD71, TER119, KLRG1, and CD41.

b. Clustering and MST representation of stained splenic cells, from Supplementary Figure 5a, above. The identical MST to that in main Figure 4b is provided for convenience and comparison to the biaxial plots here. T cell markers that co-express on same cell subsets (TCRb, CD3, CD4, CD8, and CXCR3) are circled with a light brown line. B cell markers that co-express (B220, IgM, CD19) as well as MHC and CD38 known to express on B cells are circled with a light blue line. Erythroid marker co-expression of TER119, CD49D, and CD71 is circled with a light grey line.

Supplementary Figure 6

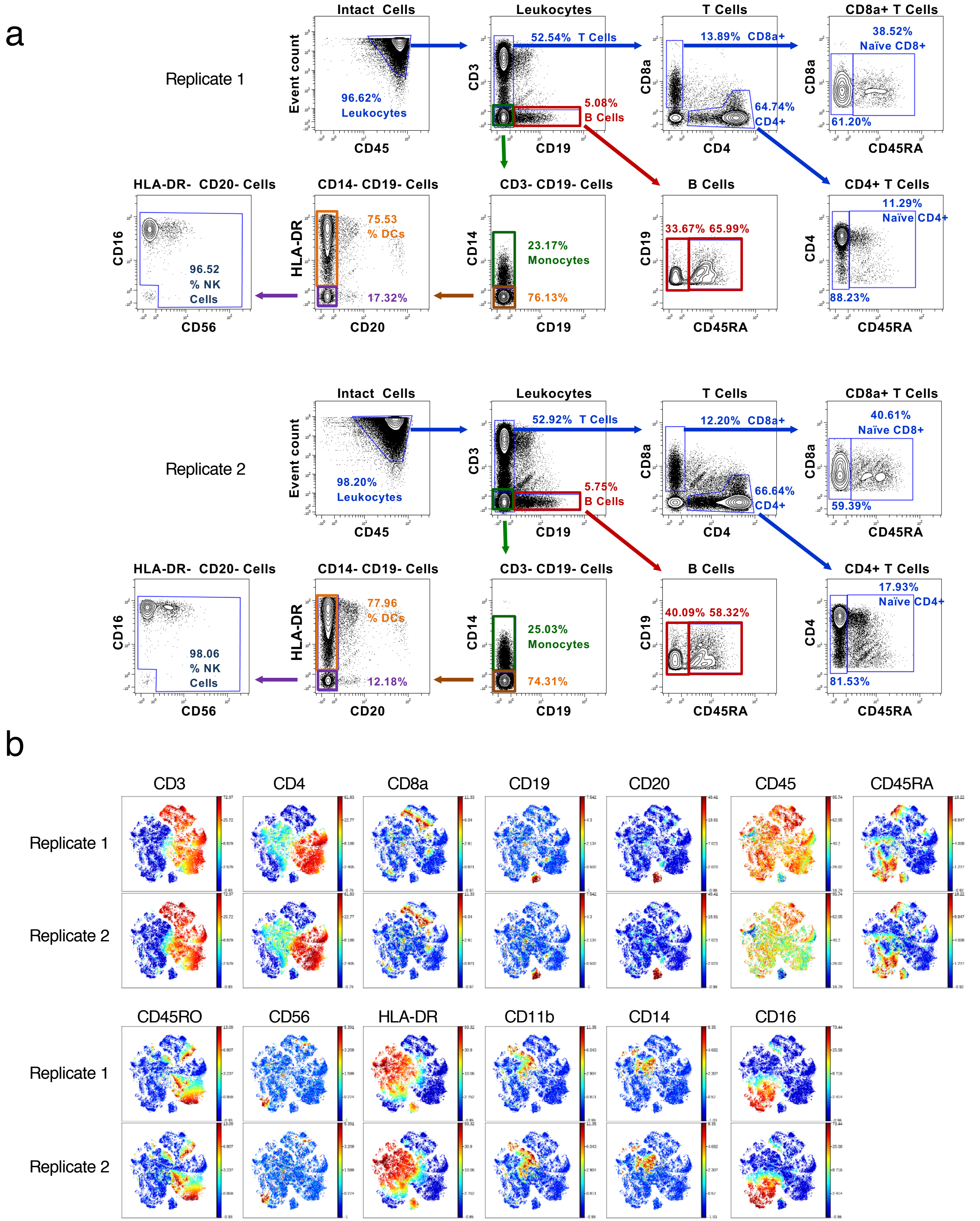

Supplementary Figure 6

a. Bivariate gating of QBC biological replicates of a 13 anti-human antibody panel stained on healthy human PBMCs. TotalSeq-B antibodies used in the panel were CD3, CD4, CD8a, CD19, CD20, CD45, CD45RA, CD45RO, CD56, HLA-DR, CD11b, CD14, and CD16. 47,081 (replicate 1) and 92,016 (replicate 2) cells were identified by data analysis.

b. viSNE plots. Data from the biological replicates in Supplementary Figure 6a were clustered via viSNE using 12 out of the 13 markers. CD45 was left out of the clustering analysis to observe how the other leukocyte markers correlated with each other.

Supplementary Figure 7

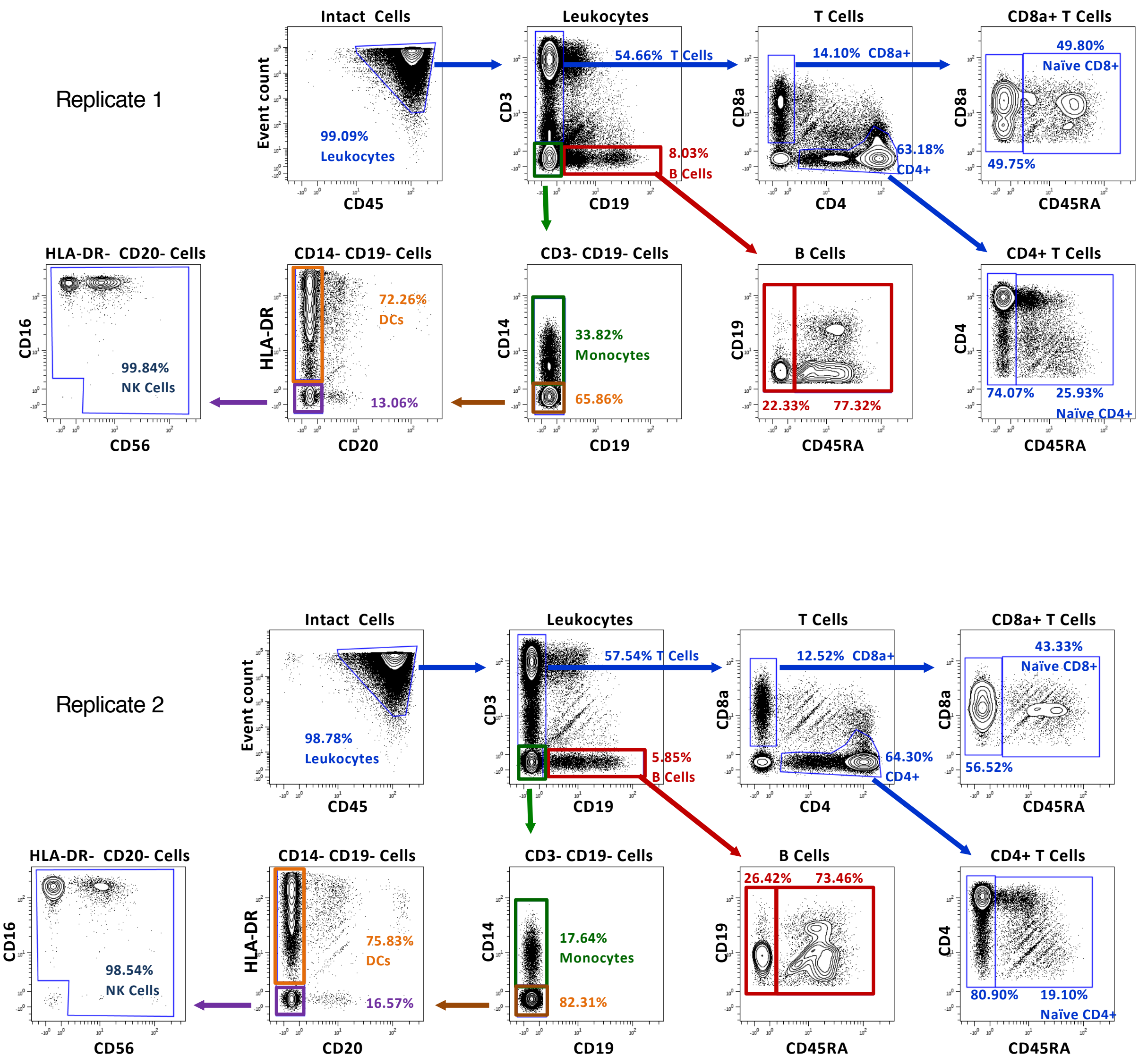

Bivariate gating of QBC biological replicates of a 32 anti-human antibody panel stained on healthy human PBMCs. TotalSeq-B antibodies used in the panel were CD3, CD4, CD8a, TIGIT, CD274, CD27, CD28, CD25, CD137, CD19, CD20, CD45, CD45RA, CD45RO, CD56, CD69, HLA-DR, CD11b, CD14, CD197, CD15, CD16, CD62L, CD86, CD80, CD127, CD278, CD279, CD335, IgG1, IgG2a, and IgG2b. 92,788 (replicate 1) and 82,478 (replicate 2) cells were identified by data analysis.

Supplementary Figure 8

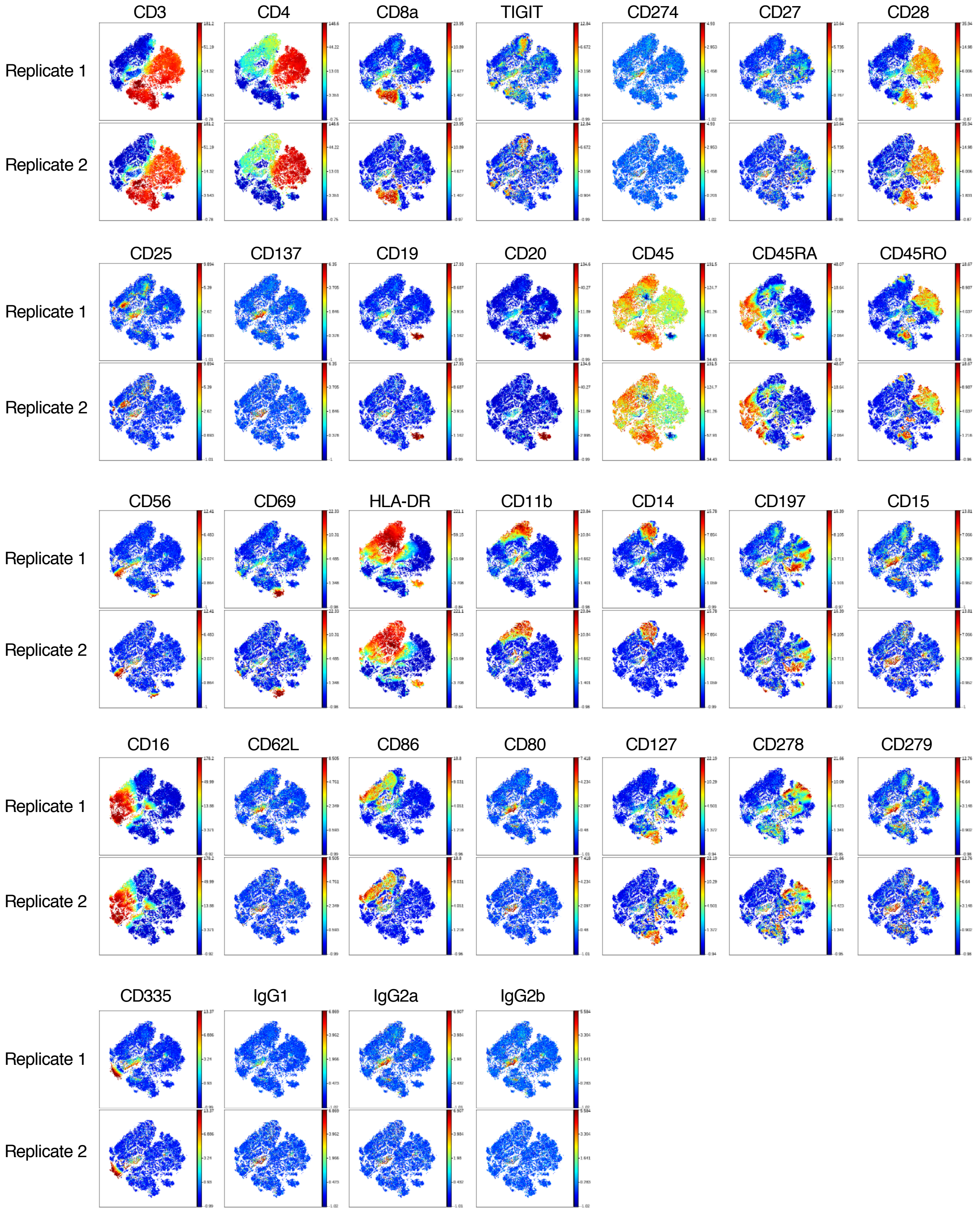

viSNE plots of QBC biological replicates of a 32 anti-human antibody panel stained on healthy human PBMCs. Data from the experimental replicates in Supplementary Figure 7 were clustered via viSNE using 28 out of the 32 markers. CD45 and the three anti-mouse IgG controls were left out of the clustering analysis to observe how the other anti-human leukocyte markers correlated with each other.

Supplementary Figure 9

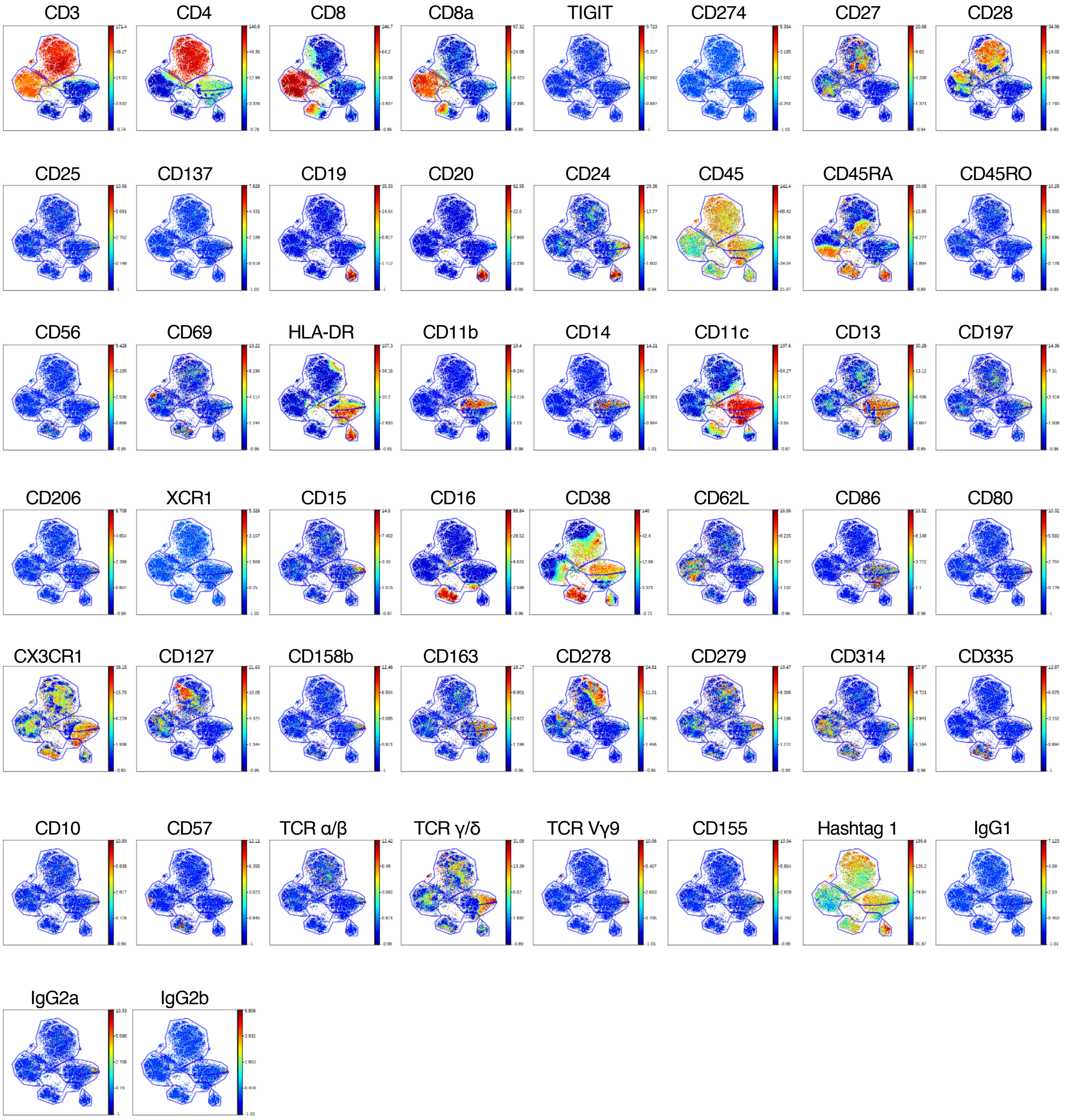

viSNE plots for QBC experiment with 50 anti-human antibody panel on healthy human PBMCs. Complete set of viSNE plots for data in Figure 6b.

Supplementary Figure 10

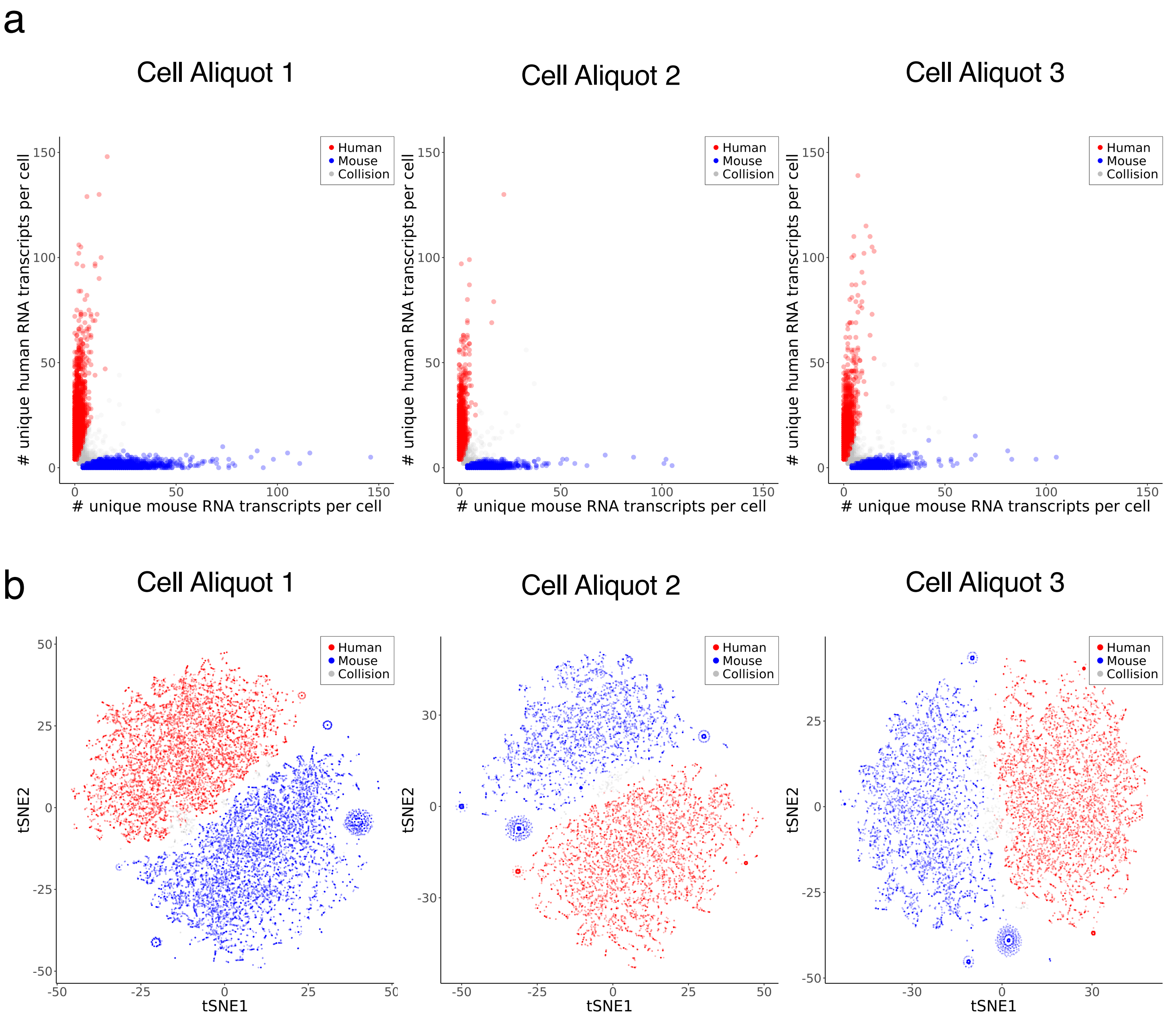

a. A 50:50 mixture of mouse BW5147 and human Nalm6 cells were processed by targeted Ins-RT-QBC. After split-pool single-cell barcoding, three aliquots of ~50,000 cells each were processed for cell lysis, second strand synthesis, PCR for library prep, and sequencing. Scatter plots of the number of unique RNA transcripts of each species type per cell for the three aliquots. Species type was assigned by color if ≥75% of the total unique transcripts were from one of the species, otherwise the cells were labeled as collision. Plots represent 25,603 cells (cell aliquot 1), 12,499 cells (cell aliquot 2), 15,148 cells (cell aliquot 3).

b. tSNE clustering of the mixtures from Supplementary Figure 10a. Normalized data were visualized by t-SNE. Cells were colored as human (red), mouse (blue) and collision (grey) based on the cluster-assignment inferred from the number of unique RNA transcripts per cell as described in Supplementary Figure 10a.

Supplementary Figure 11

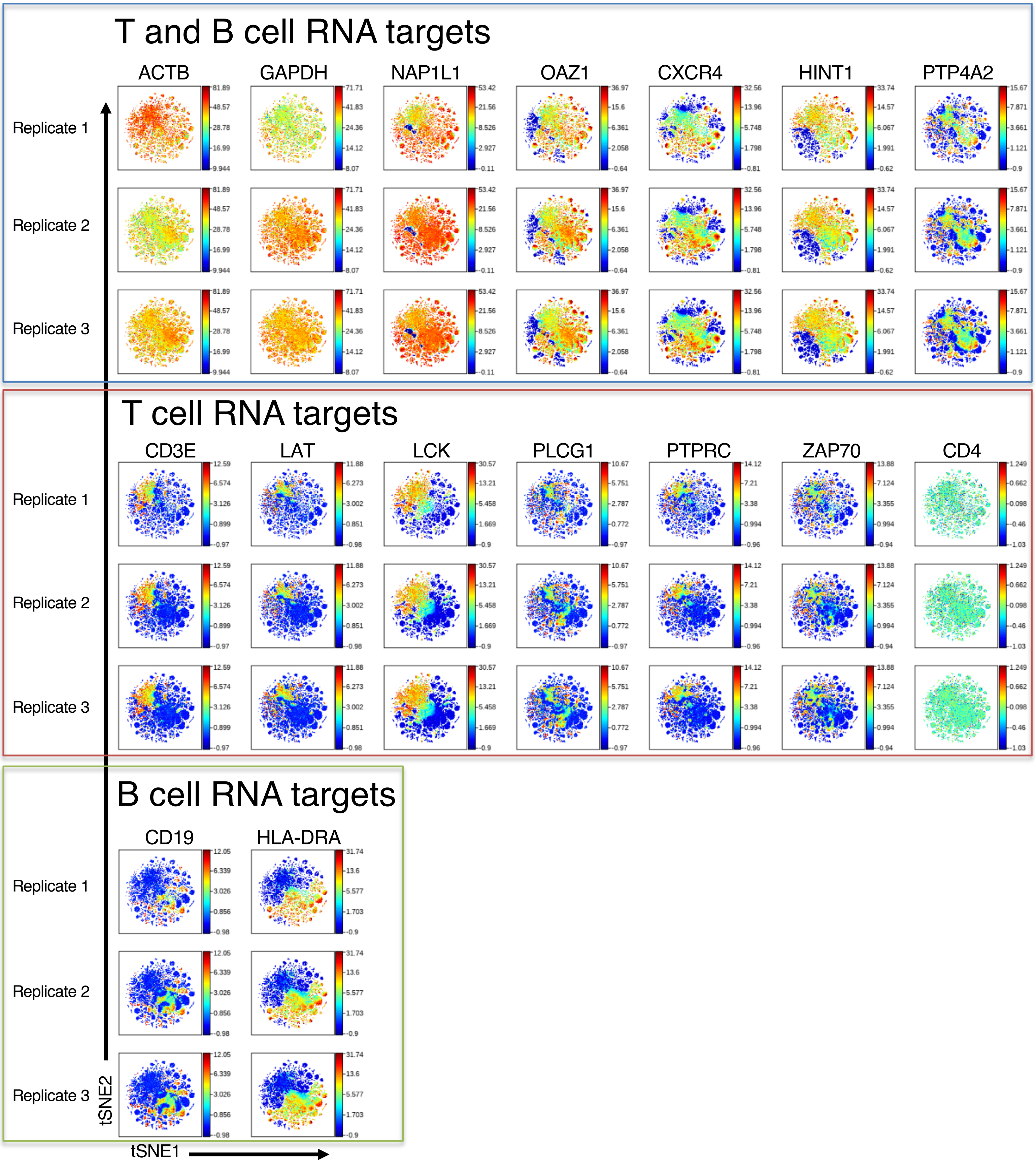

Supplementary Figure 11

Expression of T-cell and B-cell signaling proteins demonstrated via viSNE plots of 50:50 mixtures of Jurkat and Nalm6 cell lines. Three Ins-RT-QBC biological replicates. The samples were sequenced on a NextSeq instrument resulting in ~71M, ~57M, ~29M raw reads and single-cell deconvolution analysis yielded 80,808, 25,473, 51,760 cells, respectively. There was an average of 43, 35, and 43 unique transcripts per cell for each replicate, respectively. Data was rendered via viSNE using NAP1L1, OAZ1, CXCR4, HINT1, PTP4A2, CD3E, LAT, LCK, PLCG1, PTPRC, ZAP70, CD4m CD19, and HLA-DRA as selected channels to organize the viSNE plots. The housekeeping markers GAPDH, ACTB were deliberately excluded from the viSNE run to view how the expression of the other markers correlated with each other. Expression of the RNA targets expected to be expressed in both cell types of the mixture were observed to be present across the regions of the viSNE plots. T cell only RNA targets were obsetrved to co-express on the upper left region of the viSNE plots while CD19 and HLA-DRA B cell RNA targets were co-expressed in the lower right region of the viSNA plots. Anti-expression of the T and B cell markers in clearly visible. Plots represent 25,473 cells (replicate 1), 51,760 cells (replicate 2), and 80,808 cells (replicate 3).

Supplementary Figure 12

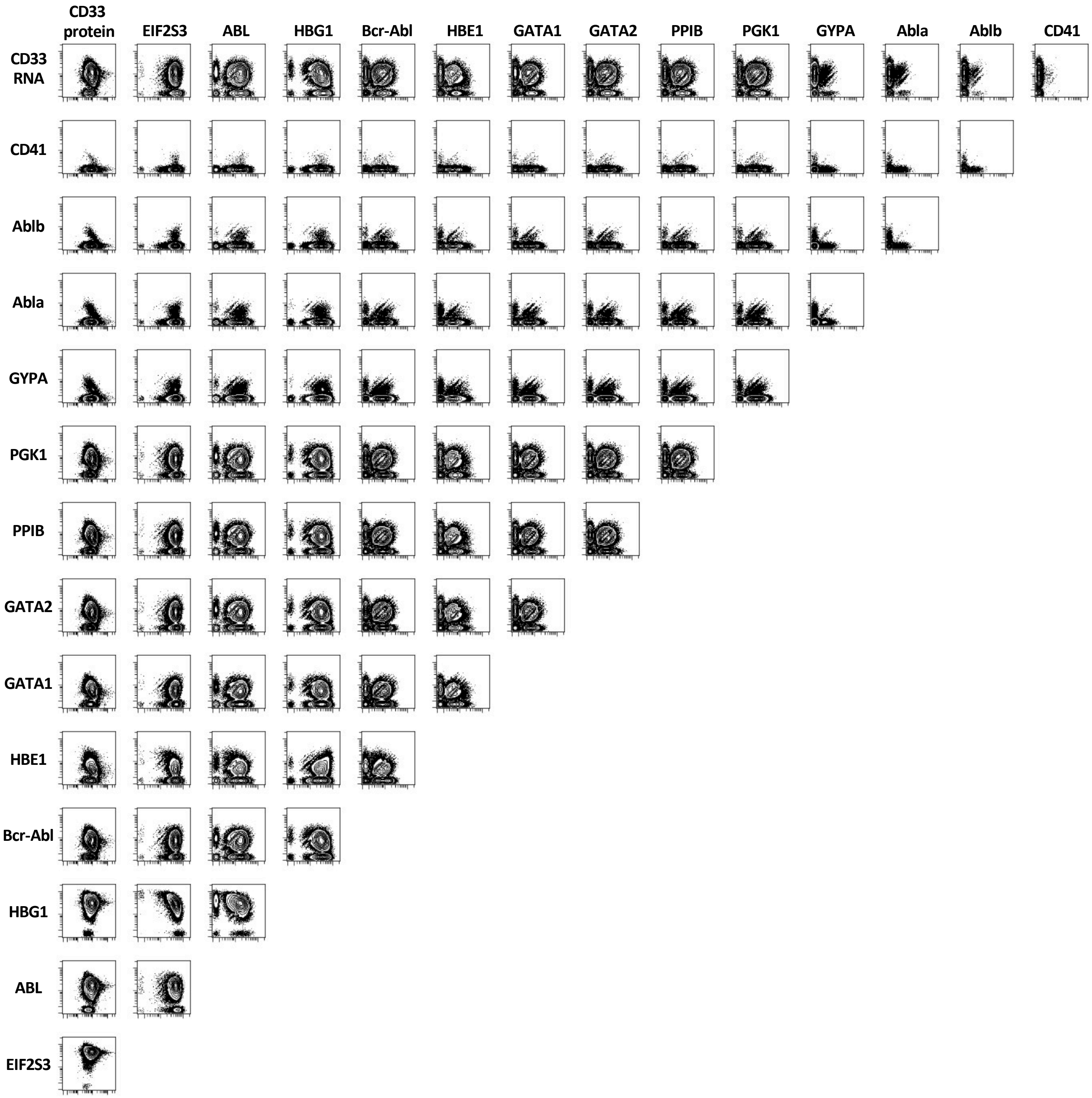

Pairwise biaxial plots for data in Figure 9a. Primers for ABL, CD33, EIF2S3, PPIB, HBE1, HBG1, GATA1, GATA2, BCR-ABL, ABLa, Ablb, GYPA, and CD41 were used for this InsRT-QBC experiment. Cells were stained with anti-CD33 antibody. Plots represent 56,950 cells.

Supplementary Figure 13

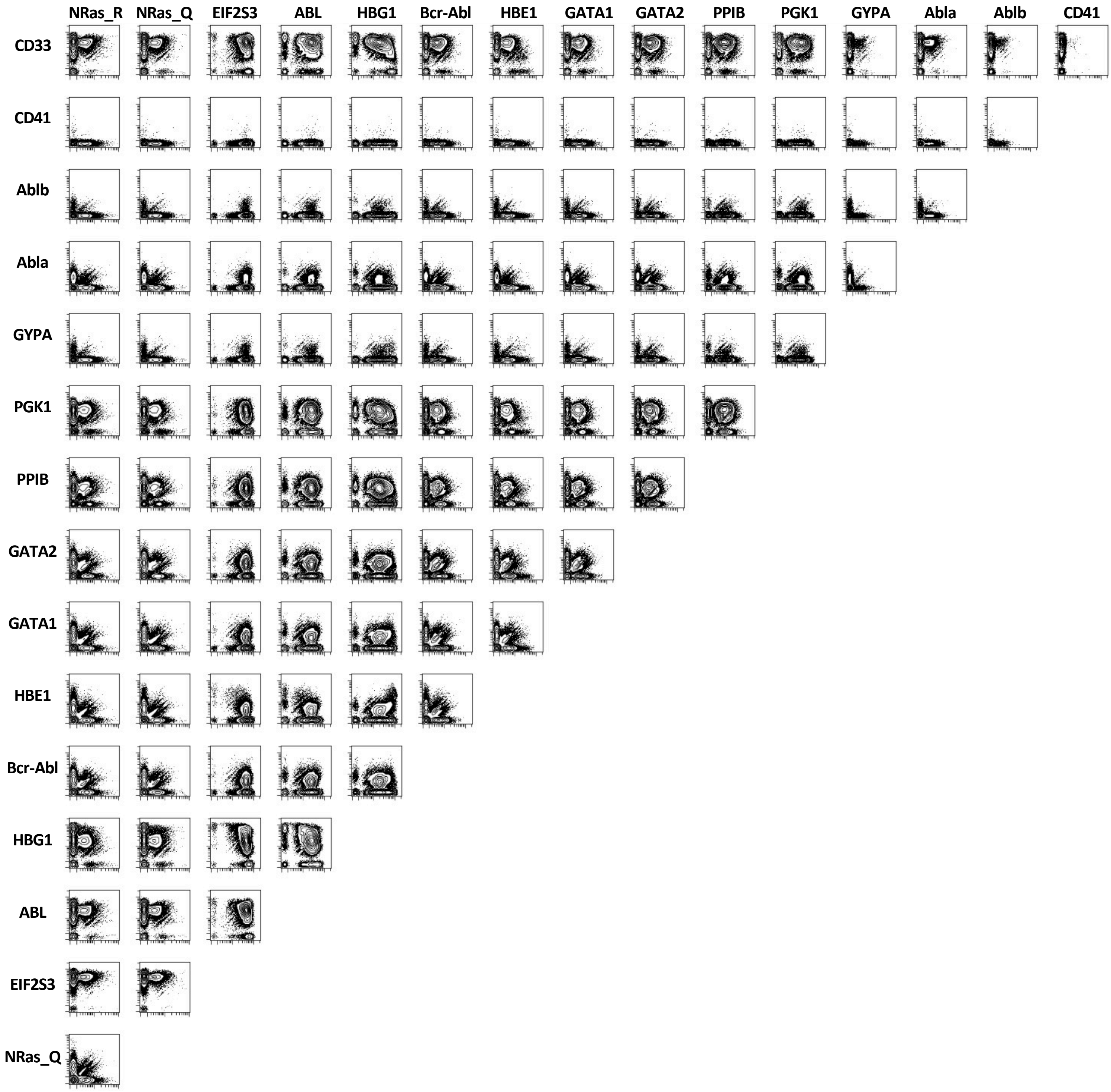

Pairwise biaxial plots for data in Figures 9b-c. A 50%:50% mixture of K562 cells and SK-MEL-2 cells were analyzed for single-cell expression of 15 RNA transcripts. Plots represent 54,085 cells.

Supplementary Figure 14

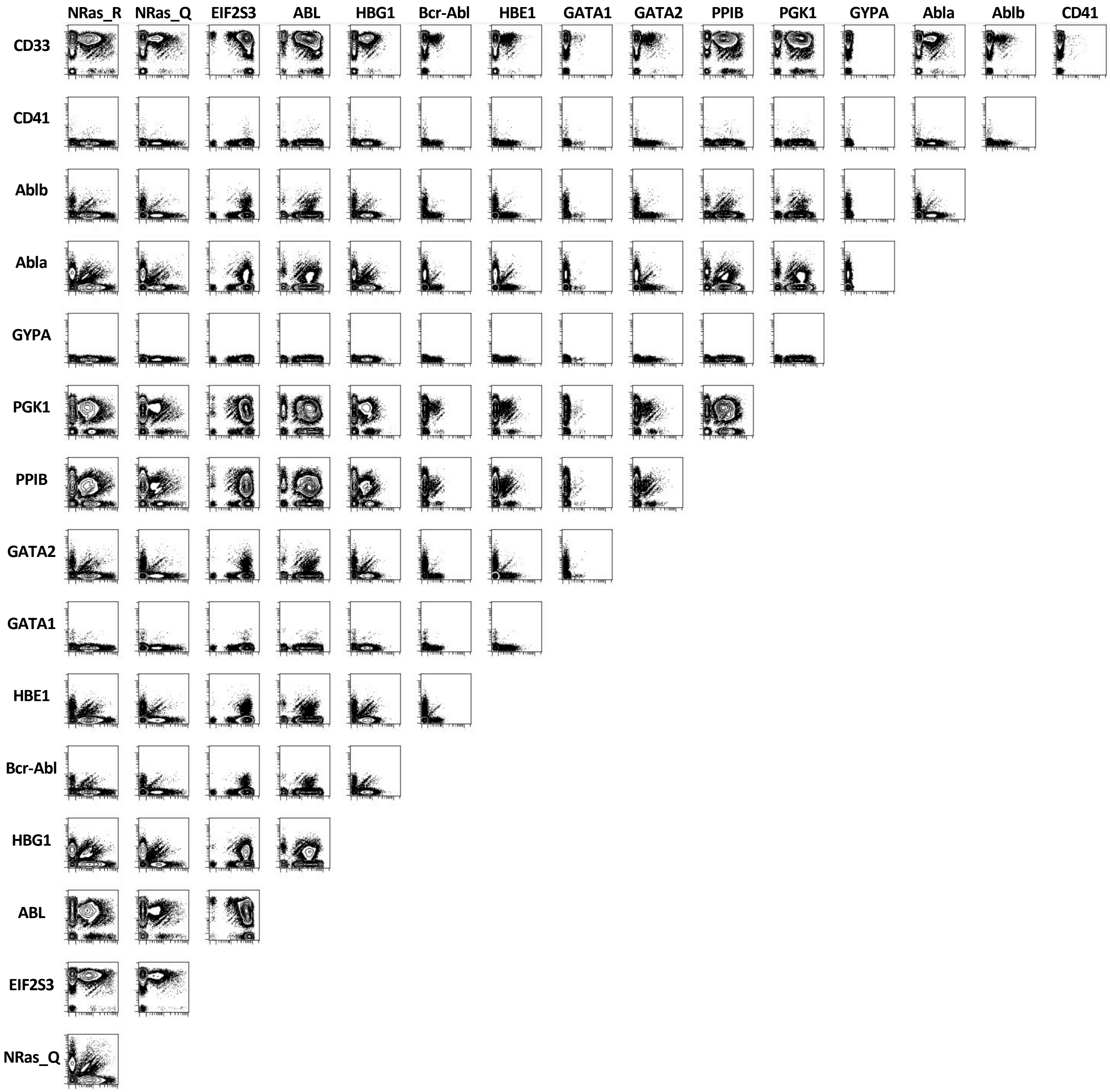

Pairwise biaxial plots for data in Figure 9b-c. A 5%:95% mixture of K562 cells and SK-MEL-2 cells were analyzed for single-cell expression of 15 RNA transcripts. Plots represent 60,754 cells.

Supplementary Figure 15

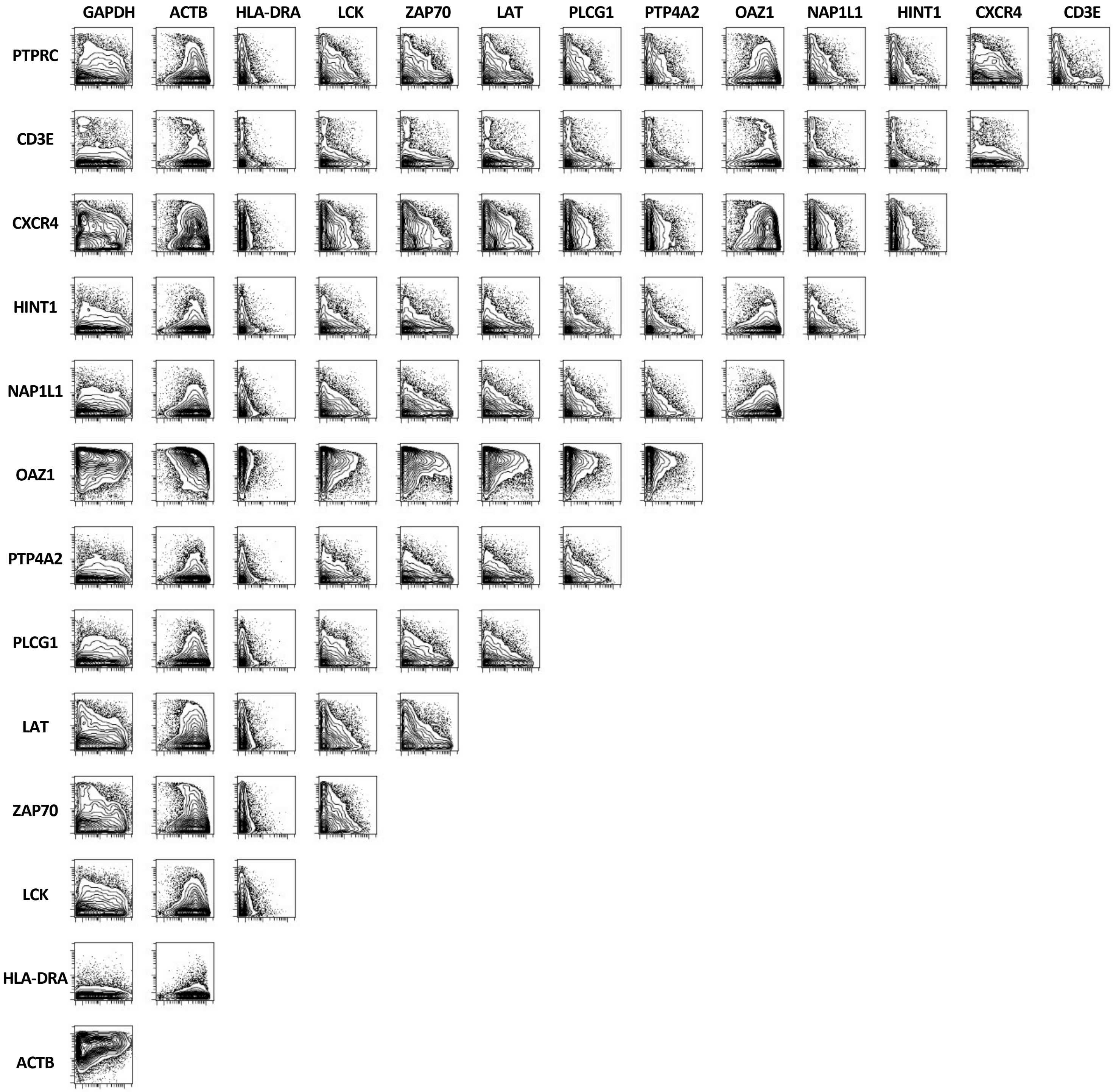

Single-cell mRNA expression measured by quantum barcoding paired with SNAIL RNA amplification. Pairwise biaxial plots for data in Figure 10b. Human PBMC were prepared for SNAIL amplification against mRNA transcripts for GAPDH, HLA-DRA, PTPRC, CD3E, LAT, PLCG1, LCK, ZAP70, NAP1L1, OAZ1, PTP4A2, CDCR4, HINT1, and ACTB. Plots represent 21,143 cells.

Supplementary Figure 16

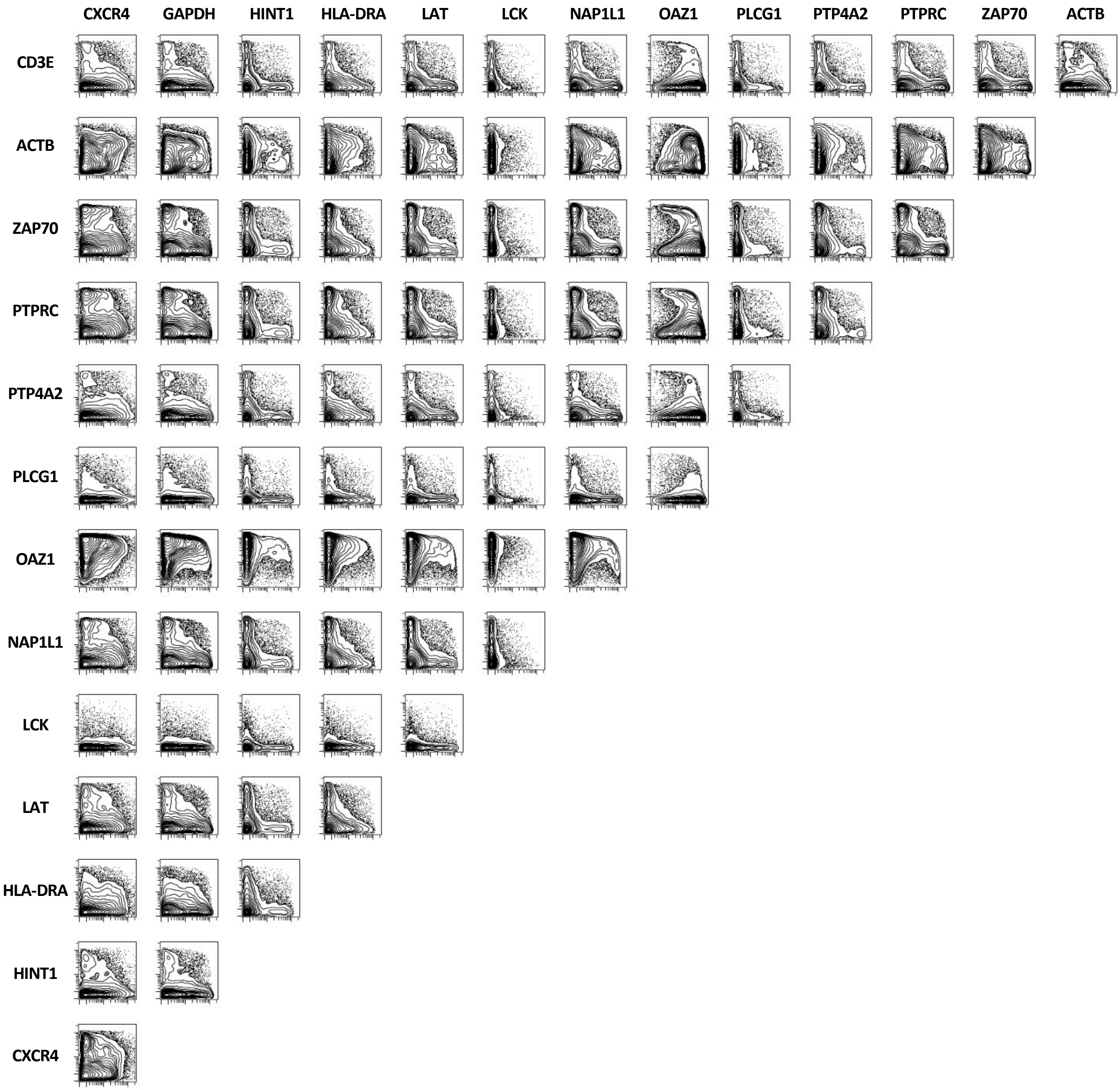

Single-cell mRNA expression measured by quantum barcoding paired with SNAIL RNA amplification. Pairwise biaxial plots for biological replicate of data from Figure 10b. Human PBMC were prepared for SNAIL amplification against mRNA transcripts for GAPDH, HLA-DRA, PTPRC, CD3E, LAT, PLCG1, LCK, ZAP70, NAP1L1, OAZ1, PTP4A2, CDCR4, HINT1, and ACTB. Plots represent 23,078 cells.

Supplementary Figure 17

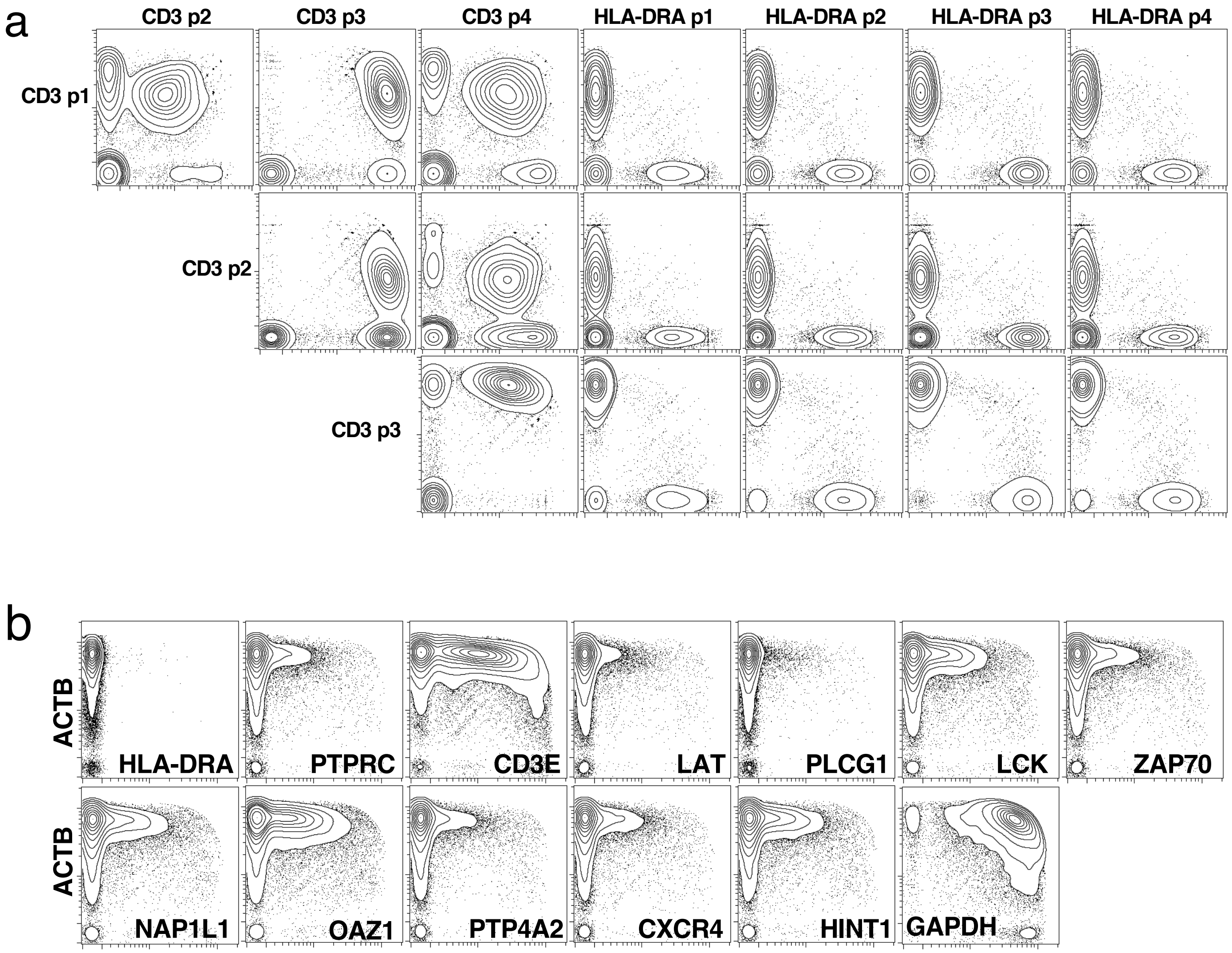

a. A 50:50 mixture of Jurkat and Nalm6 cells shouws single-cell mRNA expression from four CD3 padlock primer pairs (CD3 p1-p4) in Jurkat cells and four HLA-DRA padlock primer pairs (HLA-DRA p1-p4) in Nalm 6 cells. CD3 or HLA-DRA primer pairs were amplified by SNAIL separately in Jurkat or Nalm 6 cells, respectively. Cell lines were combined for quantum barcoding and sequencing. Plots represent 10,589 cells.
b. Single-cell expression analysis of 14 mRNA transcripts in Jurkat cell line. mRNA primer pairs were amplified by SNAIL in Jurkat cells. 27,000 cells detected by QBC. mRNA targets were: HLA-DRA, CD3E, PTPRC, LAT, PLCG1, LCK, ZAP70, NAP1L1, OAZ1, PTP4A2, CXCR4, HINT1, ACTB, and GAPDH. Plots represent 33,808 cells.

Supplementary Table 1. Table for Duplicate Barcode Calculations

Bday Problem	GoalSeek	5	# Cells	# Bar Codes	Repeats	% Error	16 SCs	24 SCs	34 SCs	60 SCs	96 SCs
							16	24	34	60	96
							# Rounds	# Rounds	# Rounds	# Rounds	# Rounds
			50,000,000	483,166,751	2,500,118	5.0	7.2	6.3	5.7	4.9	4.4
			500,000	4,831,527	25,002	5.0	5.6	4.8	4.4	3.8	3.4
			100,000	966,330	5,000	5.0	5.0	4.3	3.9	3.4	3.0
			50,000	483,181	2,500	5.0	4.7	4.1	3.7	3.2	2.9
			10,000	96,631	500	5.0	4.1	3.6	3.3	2.8	2.5
		6	1,000	9,655	50	5.0	3.3	2.9	2.6	2.2	2.0
			100	957	5	5.0	2.5	2.2	1.9	1.7	1.5

Bday Problem	GoalSeek	1	# Cells	# Bar Codes	Repeats	% Error	16 SCs	24 SCs	34 SCs	60 SCs	96 SCs
							16	24	34	60	96
							# Rounds	# Rounds	# Rounds	# Rounds	# Rounds
			50,000,000	2,482,516,613	500,162	1.0	7.8	6.8	6.1	5.3	4.7
			500,000	24,819,175	5,003	1.0	6.1	5.4	4.8	4.2	3.7
			100,000	4,962,560	1,001	1.0	5.6	4.9	4.4	3.8	3.4
			50,000	2,482,866	500	1.0	5.3	4.6	4.2	3.6	3.2
			10,000	496,413	100	1.0	4.7	4.1	3.7	3.2	2.9
		2	1,000	49,625	10	1.0	3.9	3.4	3.1	2.6	2.4
			100	4,918	1	1.0	3.1	2.7	2.4	2.1	1.9

Supplementary Table 2. Patents associated with this study.

Application or patent Serial No	Country	Status	Case Title	Inventor	Applicant
2670863	EP	Granted	METHODS OF IDENTIFYING MULTIPLE EPITOPES IN CELLS	Garry P. Nolan	F. Hoffman La Roche, AG
ZL201280016828.5	CN	Granted	METHODS OF IDENTIFYING MULTIPLE EPITOPES IN CELLS	Garry P. Nolan	F. Hoffman La Roche, AG
2670863	CH	Granted	METHODS OF IDENTIFYING MULTIPLE EPITOPES IN CELLS	Garry P. Nolan	F. Hoffman La Roche, AG
2670863	DE	Granted	METHODS OF IDENTIFYING MULTIPLE EPITOPES IN CELLS	Garry P. Nolan	Roche Diagnostics, GmbH
2670863	ES	Granted	METHODS OF IDENTIFYING MULTIPLE EPITOPES IN CELLS	Garry P. Nolan	F. Hoffman La Roche, AG
2670863	FR	Granted	METHODS OF IDENTIFYING MULTIPLE EPITOPES IN CELLS	Garry P. Nolan	F. Hoffman La Roche, AG
2670863	GB	Granted	METHODS OF IDENTIFYING MULTIPLE EPITOPES IN CELLS	Garry P. Nolan	F. Hoffman La Roche, AG
2670863	IT	Granted	METHODS OF IDENTIFYING MULTIPLE EPITOPES IN CELLS	Garry P. Nolan	F. Hoffman La Roche, AG
6069224	JP	Granted	METHODS OF IDENTIFYING MULTIPLE EPITOPES IN CELLS	Garry P. Nolan	F. Hoffman La Roche, AG
10144950	US	Granted	METHODS OF IDENTIFYING MULTIPLE EPITOPES IN CELLS	Garry P. Nolan	Roche Sequencing Solutions, Inc.
15/597917	US	Pending	METHODS OF IDENTIFYING MULTIPLE EPITOPES IN CELLS	Garry P. Nolan	Roche Sequencing Solutions, Inc
16/163486	US	Pending	METHODS OF IDENTIFYING MULTIPLE EPITOPES IN CELLS	Garry P. Nolan, Felice Alessio-Bava,	Roche Sequencing Solutions, Inc. Stanford University

Application or patent Serial No	Country	Status	Case Title	Inventor	Applicant
				Yuri Goltsev, Maeve O'Hullachain, Nikolay Samusik	
16/147250	US	Pending	METHODS OF IDENTIFYING MULTIPLE EPITOPES IN CELLS	Garry P. Nolan	Roche Sequencing Solutions, Inc
2882868	EP	Granted	INCREASING DYNAMIC RANGE FOR IDENTIFYING MULTIPLE EPITOPES IN CELLS	Garry P. Nolan	F. Hoffman La Roche, AG
ZL201380052460.2	CN	Granted	INCREASING DYNAMIC RANGE FOR IDENTIFYING MULTIPLE EPITOPES IN CELLS	Garry P. Nolan	F. Hoffman La Roche, AG
2882868	CH	Granted	INCREASING DYNAMIC RANGE FOR IDENTIFYING MULTIPLE EPITOPES IN CELLS	Garry P. Nolan	F. Hoffman La Roche, AG
2882868	DE	Granted	INCREASING DYNAMIC RANGE FOR IDENTIFYING MULTIPLE EPITOPES IN CELLS	Garry P. Nolan	Roche Diagnostics, GmbH
19188905.4	EP	Pending	INCREASING DYNAMIC RANGE FOR IDENTIFYING MULTIPLE EPITOPES IN CELLS	Garry P. Nolan	F. Hoffman La Roche, AG
2882868	FR	Granted	INCREASING DYNAMIC RANGE FOR IDENTIFYING MULTIPLE EPITOPES IN CELLS	Garry P. Nolan	F. Hoffman La Roche, AG
2882868	GB	Granted	INCREASING DYNAMIC RANGE FOR IDENTIFYING MULTIPLE EPITOPES IN CELLS	Garry P. Nolan	F. Hoffman La Roche, AG
6525872	JP	Granted	INCREASING DYNAMIC RANGE FOR IDENTIFYING MULTIPLE EPITOPES IN CELLS	Garry P. Nolan	F. Hoffman La Roche, AG
2017-248778	JP	Pending	INCREASING DYNAMIC RANGE FOR IDENTIFYING MULTIPLE EPITOPES IN CELLS	Garry P. Nolan	F. Hoffman La Roche, AG
10174310	US	Granted	INCREASING DYNAMIC RANGE FOR IDENTIFYING MULTIPLE EPITOPES IN CELLS	Garry P. Nolan	Roche Sequencing Solutions, Inc
16/182491	US	Pending	INCREASING DYNAMIC RANGE FOR	Garry P. Nolan	Roche Sequencing Solutions,

Application or patent Serial No	Country	Status	Case Title	Inventor	Applicant
			IDENTIFYING MULTIPLE EPITOPES IN CELLS		Inc
16/691493	US	Pending	INCREASING DYNAMIC RANGE FOR IDENTIFYING MULTIPLE EPITOPES IN CELLS	Garry P. Nolan, Maeve O'Hullachain, Carolina Dallett, Sedide Ozturk, Sri Paladugu, Florian Rubelt, Razika Hussein	Roche Sequencing Solutions, Inc
ZL201580069538.0	CN	Granted	METHODS FOR IDENTIFYING MULTIPLE EPITOPES IN SELECTED SUB-POPULATIONS OF CELLS	Garry P. Nolan	F. Hoffman La Roche, AG
15871277.8	EP	Pending	METHODS FOR IDENTIFYING MULTIPLE EPITOPES IN SELECTED SUB-POPULATIONS OF CELLS	Garry P. Nolan	F. Hoffman La Roche, AG
2017-532096	JP	Pending	METHODS FOR IDENTIFYING MULTIPLE EPITOPES IN SELECTED SUB-POPULATIONS OF CELLS	Garry P. Nolan	F. Hoffman La Roche, AG
15/525876	US	Pending	METHODS FOR IDENTIFYING MULTIPLE EPITOPES IN SELECTED SUB-POPULATIONS OF CELLS	Garry P. Nolan	Roche Sequencing Solutions, Inc
