# Supplementary material for: Ultra-high throughput single-cell analysis of proteins and RNAs by split-pool synthesis
Source: Commun Biol. 2020 May 7;3:213. doi: 10.1038/s42003-020-0896-2 (PMC7205613; doi:10.1038/s42003-020-0896-2)
Supplement: Supplementary file 2 — Description of Additional Supplementary Items [file 42003_2020_896_MOESM2_ESM.pdf]

1. **Supplementary Data 1**. QBC Sequences
2. **Supplementary Data 2**. AHCA Sequences
3. **Supplementary Data 3**. Subcode 1 (SC1), Subcode N (SCN), and Subcode 2(SC2) sequences
4. **Supplementary Data 4**. Subcode 3 (SC3) sequences
5. **Supplementary Data 5**. RNA-specific reverse transcription primer sequences panel for Figure 7b-c
6. **Supplementary Data 6**. RNA-specific reverse transcription primer sequences panel for Figure 8
7. **Supplementary Data 7**. RNA-specific reverse transcription primer sequences panel for Figure 9
8. **Supplementary Data 8**. RNA-specific second strand synthesis primer sequences panel for Figure 7b-c
9. **Supplementary Data 9**. RNA-specific second strand synthesis primer sequences panel for Figure 8
10. **Supplementary Data 10**. RNA-specific second strand synthesis primer sequences panel for Figure 9
11. **Supplementary Data 11**. SNAIL Probes
12. **Supplementary Data 12**. Sequence analysis metrics.
13. **Supplementary Data 13**. Figure data files and cytometry biaxial plot settings
