# Supplementary material for: Ultra-high throughput single-cell analysis of proteins and RNAs by split-pool synthesis
Source: Commun Biol. 2020 May 7;3:213. doi: 10.1038/s42003-020-0896-2 (PMC7205613; doi:10.1038/s42003-020-0896-2)
Supplement: Supplementary file 4 — Reporting Summary [file 42003_2020_896_MOESM4_ESM.pdf]

nature research

Corresponding author(s): Garry P. Nolan
Last updated by author(s): Jan 27, 2020

Reporting Summary

Nature Research wishes to improve the reproducibility of the work that we publish. This form provides structure for consistency and transparency in reporting. For further information on Nature Research policies, see Authors & Referees and the Editorial Policy Checklist.

Statistics

For all statistical analyses, confirm that the following items are present in the figure legend, table legend, main text, or Methods section.

n/a	Confirmed	
☐	☒	The exact sample size (n) for each experimental group/condition, given as a discrete number and unit of measurement
☐	☒	A statement on whether measurements were taken from distinct samples or whether the same sample was measured repeatedly
☒	☐	The statistical test(s) used AND whether they are one- or two-sided<br>*Only common tests should be described solely by name; describe more complex techniques in the Methods section.*
☒	☐	A description of all covariates tested
☐	☒	A description of any assumptions or corrections, such as tests of normality and adjustment for multiple comparisons
☐	☒	A full description of the statistical parameters including central tendency (e.g. means) or other basic estimates (e.g. regression coefficient) AND variation (e.g. standard deviation) or associated estimates of uncertainty (e.g. confidence intervals)
☒	☐	For null hypothesis testing, the test statistic (e.g. F, t, r) with confidence intervals, effect sizes, degrees of freedom and P value noted<br>*Give P values as exact values whenever suitable.*
☒	☐	For Bayesian analysis, information on the choice of priors and Markov chain Monte Carlo settings
☐	☒	For hierarchical and complex designs, identification of the appropriate level for tests and full reporting of outcomes
☒	☐	Estimates of effect sizes (e.g. Cohen's d, Pearson's r), indicating how they were calculated

Our web collection on statistics for biologists contains articles on many of the points above.

Software and code

Policy information about availability of computer code

Data collection	Data collection was done on MiSeq, NextSeq, and HiSeq Illumina instruments with the standard instrument software.
Data analysis	https://github.com/bioinform/QBC_Single_Cell_Analysis_NGS

For manuscripts utilizing custom algorithms or software that are central to the research but not yet described in published literature, software must be made available to editors/reviewers. We strongly encourage code deposition in a community repository (e.g. GitHub). See the Nature Research guidelines for submitting code & software for further information.

Data

Policy information about availability of data

All manuscripts must include a data availability statement. This statement should provide the following information, where applicable:

- Accession codes, unique identifiers, or web links for publicly available datasets
- A list of figures that have associated raw data
- A description of any restrictions on data availability

Raw sequencing data, processed data in the form of normalized and unnormalized text files, and files needed to process raw data have been deposited into the Gene Expression Omnibus with accession number GSE130784
Executable and script to process fasta to FCS can be found here:
(https://github.com/bioinform/QBC_Single_Cell_Analysis_NGS)

Field-specific reporting

Please select the one below that is the best fit for your research. If you are not sure, read the appropriate sections before making your selection.

☒ Life sciences ☐ Behavioural & social sciences ☐ Ecological, evolutionary & environmental sciences

For a reference copy of the document with all sections, see nature.com/documents/nr-reporting-summary-flat.pdf

Life sciences study design

All studies must disclose on these points even when the disclosure is negative.

Sample size	We analyzed approximately 9,000 - 90,000 cells per experiment. We based this sample size on the number of cells typically analyzed in flow cytometry experiments
Data exclusions	Exclusions included failed experiments where too few cells (below the sample size threshold stated above) were detected during analysis and where RNA expression patterns indicated non-specific binding of probes to the target transcripts.
Replication	Figures 3a, 5b, 8, 10c, and Supplementary Figures 3, 6, 7, 8, 10, 11, 16 show replication experiments where replicates were done by different operators, on different days, or processed in parallel. Replication of all experiments was not possible due to the high cost of sequencing.
Randomization	Not relevant to this study because we describe in this manuscript an experimental method tested on previously characterized sample types. We did not use randomized test and control sample groups.
Blinding	Blinding was not relevant because we did not compare test groups to control groups by our method.

Reporting for specific materials, systems and methods

We require information from authors about some types of materials, experimental systems and methods used in many studies. Here, indicate whether each material, system or method listed is relevant to your study. If you are not sure if a list item applies to your research, read the appropriate section before selecting a response.

Materials & experimental systems

n/a	Involved in the study
☐	☒ Antibodies
☐	☒ Eukaryotic cell lines
☒	☐ Palaeontology
☐	☒ Animals and other organisms
☒	☐ Human research participants
☒	☐ Clinical data

Methods

n/a	Involved in the study
☒	☐ ChIP-seq
☒	☐ Flow cytometry
☒	☐ MRI-based neuroimaging

Antibodies

Antibodies used

The following antibodies were obtained from Becton Dickinson:
B220(RA3-6B2)
CD3(17A2)
CD4(RM4-4)
CD41(MWReg30)
CD44(IM7)
CD8a(53-6.7)
Ly6d(49H4)
TCRb(H57-597)
The following antibodies were obtained from Affymetrix:
B220(RA3-6B2)
CD11b(M1/70)
CD19(1D3)
CD3(145-2C11)
CD34(RAM34)
CD38(90)
CD4(GK1.5)
CD62L(MEL-14)
CD8a(53-6.7)
CD8b(H35-17.2)
CXCR3(CXCR3-173)
IgM(II/41)

	KLRG1(2F1)<br>MHC(M5/114.15.2)<br>PCDA1(eBio927)<br>TCRb(H57-597)<br>TER119(TER-119)<br>The following antibodies were obtained from Biolegend:<br>CD33(WM53)<br>CD49d(9C10(MFR4.B))<br>Gr1(RB6-8C5)<br>IgM(RMM-1)<br>The following anti-human antibody oligo conjugates were obtained from Biolegend's TotalSeq-B catalog: CD3, CD4, CD8, CD8a, TIGIT, CD274, CD27, CD28, CD25, CD137, CD19, CD20, CD24, CD45, CD45RA, CD45RO, CD56, CD69, HLA-DR, CD11b, CD14, CD11c, CD13, CD197,CD206, XCR1, CD15, CD16, CD38, CD62L, CD86, CD80, CX3CR1, CD127, CD158b, CD163, CD278, CD279, CD314, CD335, CD10, CD57, TCR α/β, TCR γ/δ, TCR Vγ9, CD155, Hashtag 1, IgG1, IgG2a, and IgG2b.<br>The following antibodies were obtained from eBiosciences:<br>CD135(A2F10)<br>CD71(R17217)
Validation	Primary antibodies were validated by flow cytometry following conjugation to an oligonucleotide to check for binding affinity in the cell type to be used for quantum barcoding

Eukaryotic cell lines

Policy information about cell lines

Cell line source(s)	K562, SK-MEL-2, Jurkat, and Nalm-6, BW5147 cells were obtained from America Type Tissue Collection (ATCC)
Authentication	Cell lines were authenticated by sequencing
Mycoplasma contamination	Cell lines tested negative to Mycoplasma contamination
Commonly misidentified lines<br>(See ICLAC register)	*Name any commonly misidentified cell lines used in the study and provide a rationale for their use.*

Animals and other organisms

Policy information about studies involving animals; ARRIVE guidelines recommended for reporting animal research

Laboratory animals	C57/Blk6 mice, between 6-8 weeks of age
Wild animals	Study did not involve wild animals
Field-collected samples	Study did not involve field-collected samples
Ethics oversight	Stanford IACUC

Note that full information on the approval of the study protocol must also be provided in the manuscript.
